# A hypoxia risk signature for the tumor immune microenvironment evaluation and prognosis prediction in acute myeloid leukemia

**DOI:** 10.1038/s41598-021-94128-1

**Published:** 2021-07-19

**Authors:** Feng Jiang, Yan Mao, Binbin Lu, Guoping Zhou, Jimei Wang

**Affiliations:** 1 Department of Neonatology, Obstetrics and Gynecology Hospital, Fudan University, No. 419, Fangxie Road, Shanghai, 200011 China; 2 Department of Pediatrics, The First Affiliated Hospital of Nanjing Medical University, Nanjing, 210029 China

**Keywords:** Cancer, Computational biology and bioinformatics, Immunology, Risk factors

## Abstract

Acute myeloid leukemia (AML) is the most prevalent form of acute leukemia. Patients with AML often have poor clinical prognoses. Hypoxia can activate a series of immunosuppressive processes in tumors, resulting in diseases and poor clinical prognoses. However, how to evaluate the severity of hypoxia in tumor immune microenvironment remains unknown. In this study, we downloaded the profiles of RNA sequence and clinicopathological data of pediatric AML patients from Therapeutically Applicable Research to Generate Effective Treatments (TARGET) database, as well as those of AML patients from Gene Expression Omnibus (GEO). In order to explore the immune microenvironment in AML, we established a risk signature to predict clinical prognosis. Our data showed that patients with high hypoxia risk score had shorter overall survival, indicating that higher hypoxia risk scores was significantly linked to immunosuppressive microenvironment in AML. Further analysis showed that the hypoxia could be used to serve as an independent prognostic indicator for AML patients. Moreover, we found gene sets enriched in high-risk AML group participated in the carcinogenesis. In summary, the established hypoxia-related risk model could act as an independent predictor for the clinical prognosis of AML, and also reflect the response intensity of the immune microenvironment in AML.

## Introduction

Hypoxia has been reported as a marker of the microenvironment in tumors. Due to the lack of blood supply, tumor cells often exist in hypoxic environments^1^. In the microenvironment with low oxygen levels, tumor cells, different from healthy cells, often initiate a series of behaviors that finally lead to more aggressive tumor phenotypes^2,3^. Acute leukemia (AL) is the most frequent cancer in children, while pediatric acute myeloid leukemia (AML) comprises approximately 25% of pediatric AL^4^. In clinical practice, risk stratification of AML patients, which is based on the clinicopathological characteristics, enables physicians to triage patients for optimal chemotherapy regimens^5^. However, the clinical outcomes of childhood AML have been consistently inferior to childhood acute lymphoblastic leukemia (ALL), which accounts for more than half of the deaths due to acute leukemia^6^. More and more evidence has pointed out that hypoxia provides a pro-survival environment to AML cells, and protects AML cells from apoptosis^7,8^. In the bone marrow of AML patients, hypoxia has been found to partially inhibit death pathway^9^. Even though hypoxia generally promotes cell quiescence, it seems that such conditions could promote AML cell proliferation, which is also the same with results of previous studies on AML^10,11^. Hence, hypoxia is associated with tumor recurrence, anti-apoptosis, chemotherapy resistance, and decreased patient survival.

It is well recognized nowadays that hypoxia plays an essential role in pushing tumor immunosuppression, and immune escape. Previous evidence indicates that in the oxygen-depleted microenvironment, natural killer (NK) cells and T cells exhibit a state of anergy or exhaustion, which eventually results in dysfunction^12–14^. Hypoxia induces the development of suppressive cells and the release of immunosuppressive cytokines, which in turn inhibit the function of immune effector cells^15^. Up to now, biomarkers for predicting the outcomes of immunotherapy mainly include programmed death-ligand 1 (PD-L1) and programmed cell death protein 1 (PD1)^16^. However, researchers often ignore the basic problem of oxygen supply in immune microenvironment. Hence, hypoxia can be used to serve as a biomarker for predicting the immunotherapy outcomes.

It is well known that methods of exploring hypoxia in tumors are limited nowadays. In this study, we used throughput mRNA profiling data from online database to build a risk signature associated with hypoxia, which could reflect the immunosuppressive microenvironment in childhood AML patients and their clinical outcomes. Furthermore, this hypoxia risk model may provide more intuitive and rational information for physicians to treat AML patients in the future.

## Results

### Construction of a hypoxia risk model to predict the clinical prognosis of childhood AML

**Figure 1:**
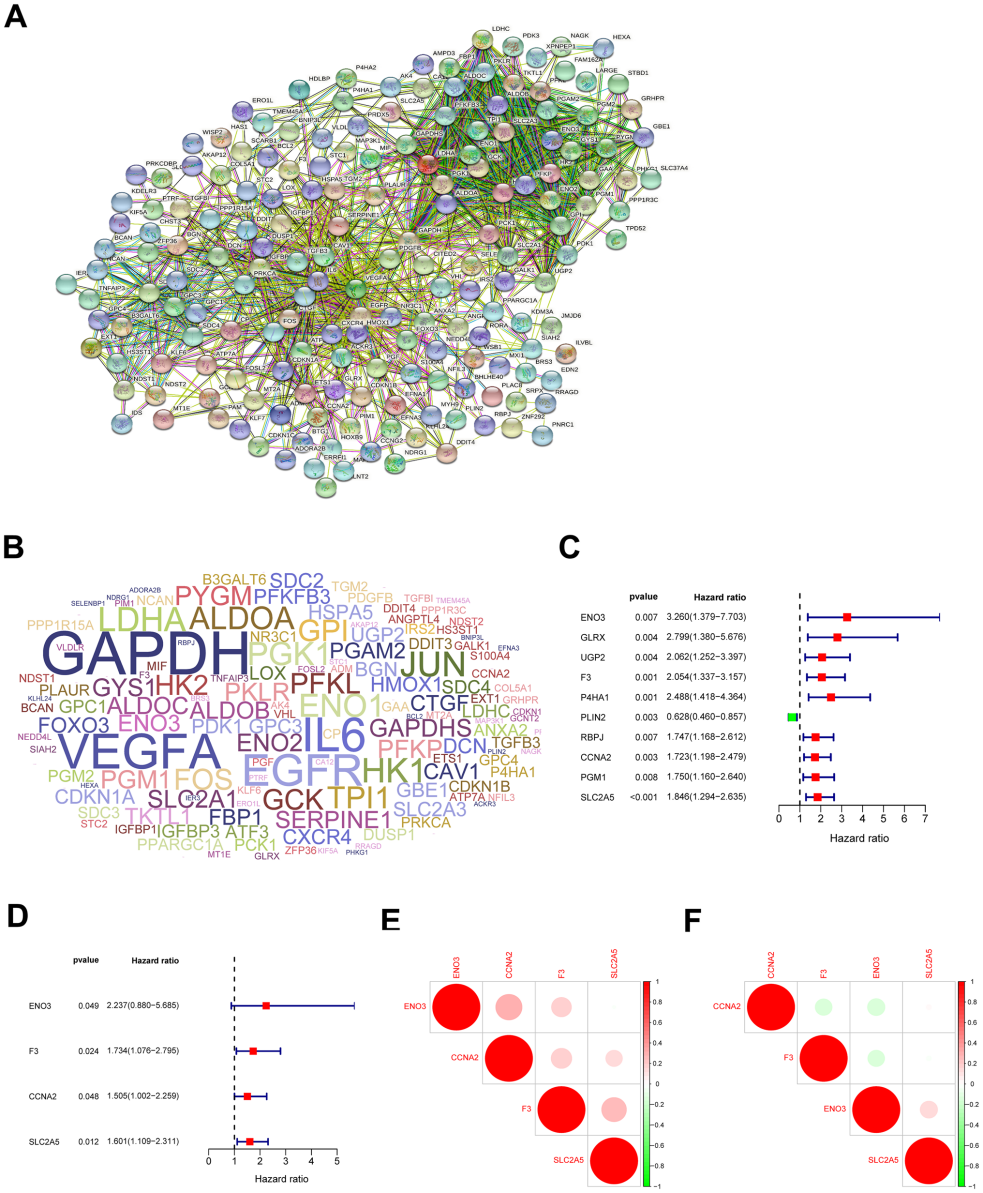
Construction of a hypoxia risk model to predict the clinical prognosis of childhood AML. (**A**) Interaction relationships among hypoxia-associated genes via STRING database; (**B**) Word frequency cloud of the top 150 hypoxia-associated genes, where the size of the gene name represented its node degree; (**C**, **D**) Screening of hypoxia-related genes by univariate and multivariate Cox regression analyses to construct a hypoxia risk model with prognosis predictive value; (**E**, **F**) Spearman correlation analysis for the identified four hypoxia-associated genes involved in the risk signature in the TARGET and GEO databases.

We downloaded the gene set correlated with hypoxia from the Gene Set Enrichment Analysis (GSEA). The gene set contained a total of 200 genes, which had been found upregulated in response to hypoxia microenvironment (Supplementary Table 1). We conducted PPI network analysis using STRING database to explore the interaction relationships among these genes (Fig. 1A). The word frequency cloud showed the identified top 150 genes with the higher node degrees (Fig. 1B & Supplementary Table 2), including GAPDH, VEGFA, IL-6, JUN, EGFR, LDHA, HK1, ENO1, PGK1, ALDOA and so on, suggesting their importance in hypoxia.

Based on the 150 genes, we developed a hypoxia risk signature using the univariate and multivariate Cox regression analyses to predict pediatric AML patients' clinical prognoses. According to the results of the univariate Cox analysis, 10 genes were found significantly associated with overall survival (OS) of pediatric AML patients (Fig. 1C). According to the multivariate Cox analysis, four genes (ENO3, F3, CCNA2 and SLC2A5) were further identified to construct the predictive model (Fig. 1D). The risk score developed was as followed: risk score = 0.81 × ENO3 + 0.55 × F3 + 0.41 × CCNA2 + 0.47 × SLC2A5. As shown in Fig. 1E & F, in both the TARGET and GEO datasets, all these four genes, closely associated with OS of pediatric AML patients (Supplementary Fig. 1), were also found significantly correlated with one another.

Hypoxia inducible factor-1a (HIF-1a) has been reported to be closely related to hypoxia. Considering that, we performed the Kaplan–Meier analysis on the expression levels of HIF-1a along with the OS of pediatric AML patients. As shown in Supplementary Fig. 2, the OS of AML patients with higher HIF-1a levels were not significantly higher than that of AML patients with lower HIF-1a levels, both in TARGET (Supplementary Fig. 2A, *P* > 0.05) and in GEO (Supplementary Fig. 2B, *P* > 0.05), suggesting that HIF-1a did not have a significant correlation with the OS of AML patients.

### Prognostic value of the hypoxia risk signature in AML patients from the TARGET and GEO databases

**Figure 2:**
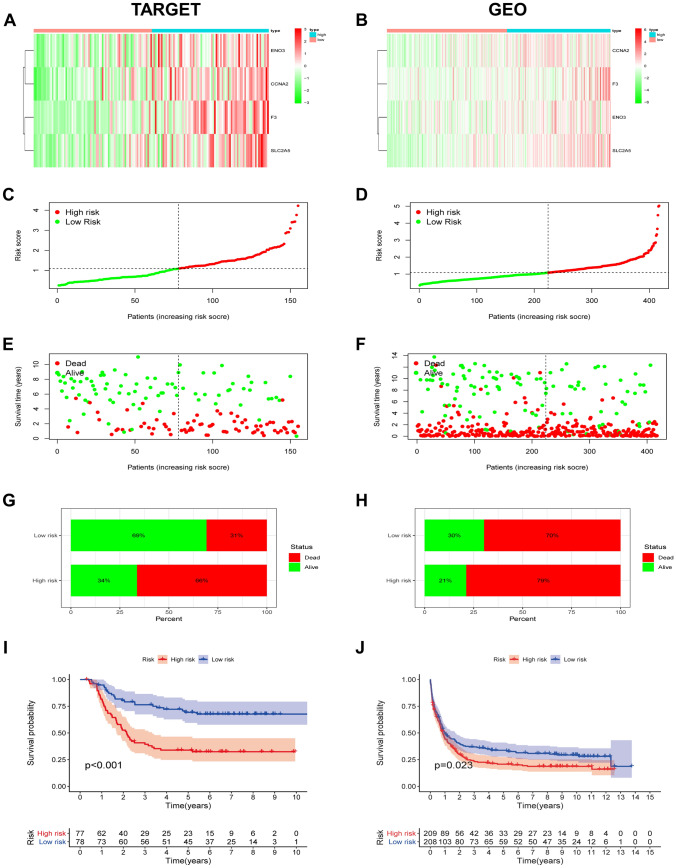
Prognostic value of the hypoxia risk signature in AML patients from the TARGET and GEO databases. (**A**) Heatmap showing the expression profiles of the four identified hypoxia genes in groups with different hypoxia risks in TARGET (https://ocg.cancer.gov/programs/target); (**B**) Heatmap showing the expression profiles of the four identified hypoxia genes in groups with different hypoxia risks in GEO (https://www.ncbi.nlm.nih.gov/geo); The heat maps in this manuscript were generated using R software (version 3.6.2; https://www.r-project.org/). (**C**, **D**) Distribution of patients’ risk scores in the two risk groups; (**E**, **F**) Distribution of patient status in the two hypoxia risk groups; (G&H) Mortality rates of the two risk groups; (**I**, **J**) Kaplan–Meier overall survival curves for AML patients in the two hypoxia risk groups.

As hypoxia has been reported to promote tumor cells to exhibit more aggressive phenotypes^17,18^, we explored the prognostic value of the developed hypoxia signature. In the TARGET database (Fig. 2A) and GEO database (Fig. 2B), the four chosen genes’ expressions were increased along with the increase of risk score, which indicated that AML patients with higher hypoxia risk tended to develop a hypoxic microenvironment when compared with other ones. Figure 2C & D revealed the risk scores of AML patients and their grouping. The AML patients in the higher hypoxia risk group had higher mortality rate than those in the lower hypoxia risk group (Fig. 2E–H). In addition, we carried out Kaplan–Meier analysis to evaluate the hypoxia risk signature's prognostic value in AML. Results showed that in the TARGET, patients with low hypoxia risk had longer survival than those with high hypoxia risk (Fig. 2I), which was validated with the GEO data (Fig. 2J). We further validate the prognostic value of the hypoxia risk signature in AML samples from TCGA database. As shown in Supplementary Fig. 3, all these results from TCGA were consistent with those from TARGET and GEO databases.

### Relationship between hypoxia genes’ expression and clinicopathological characteristics in AML

**Figure 3:**
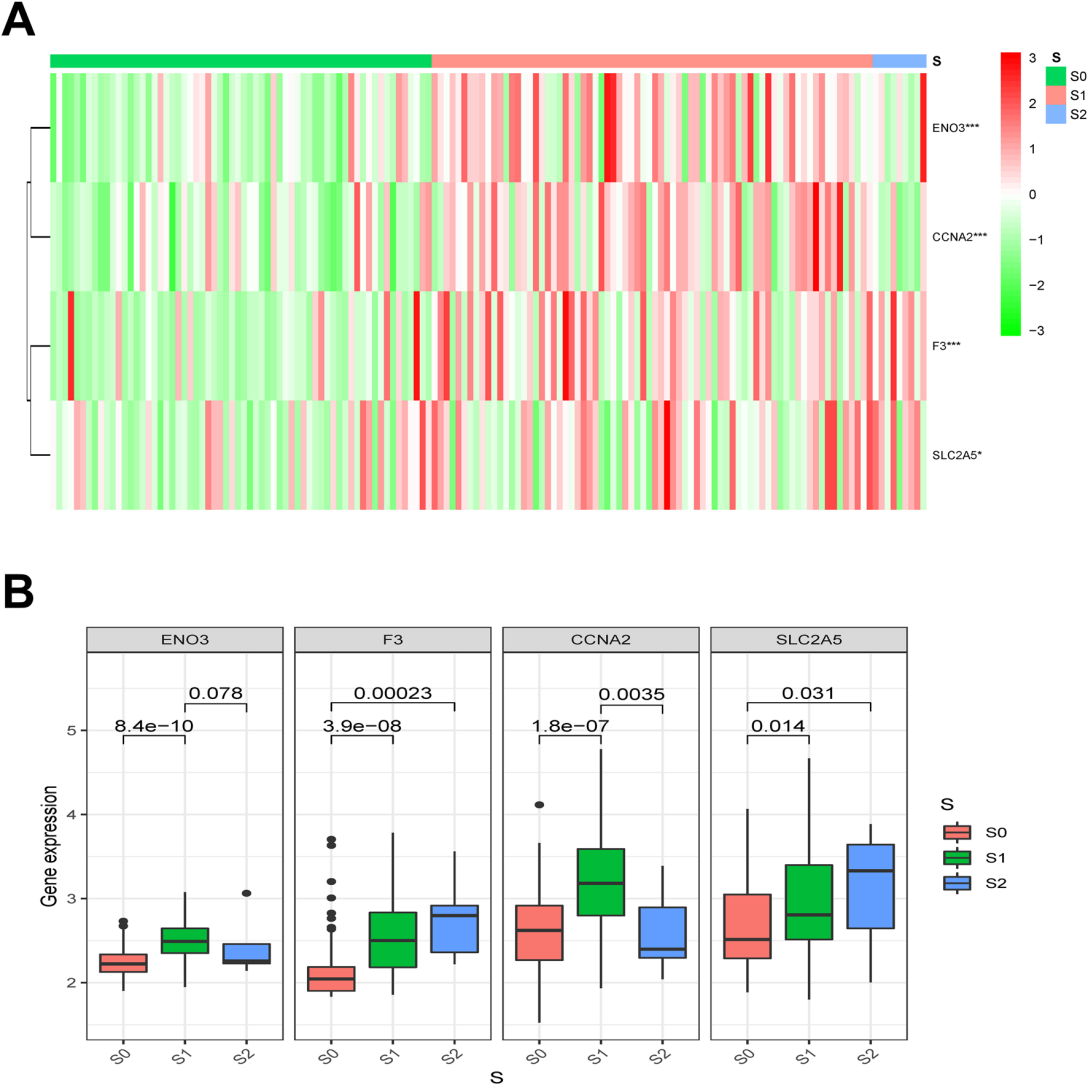
Relationship between hypoxia genes’ expression and clinicopathological characteristics in AML. (**A**) A heatmap showing the expression profiles of the four hypoxia-associated genes in different risk stratification from the TARGET (https://ocg.cancer.gov/programs/target); (**B**) The four hypoxia-related genes’ expression levels in different risk stratification in childhood AML.

Clinically, children with AML are often classified into different groups according to their clinical characteristics and MICM characteristics in the early stage of chemotherapy, which is identified as risk stratification. Risk stratification of AML patients enables physicians to triage patients for optimal therapy^19,20^. We used “S” to represent the clinical risk stratification, while “S0” represented low risk, “S1” represented intermediate risk, and “S2” represented high risk. Taking into account the biological functions of hypoxia in the occurrence and development of tumors^21–23^, the relationship between the risk stratification of AML and the four identified genes associated with hypoxia were analyzed. The heatmap showed the gene expression levels and risk stratification of AML patients in TARGET (Fig. 3A), showing that the expression of the four genes significantly increased in higher risk stratification. Quantitative analyses further confirmed the significant relationship between the risk stratification and the four genes’ expression levels (Fig. 3B), showing that the four genes’ expression levels were elevated along with the increased of risk stratification.

### Efficiency of the hypoxia risk signature in prognostic prediction in AML

**Figure 4:**
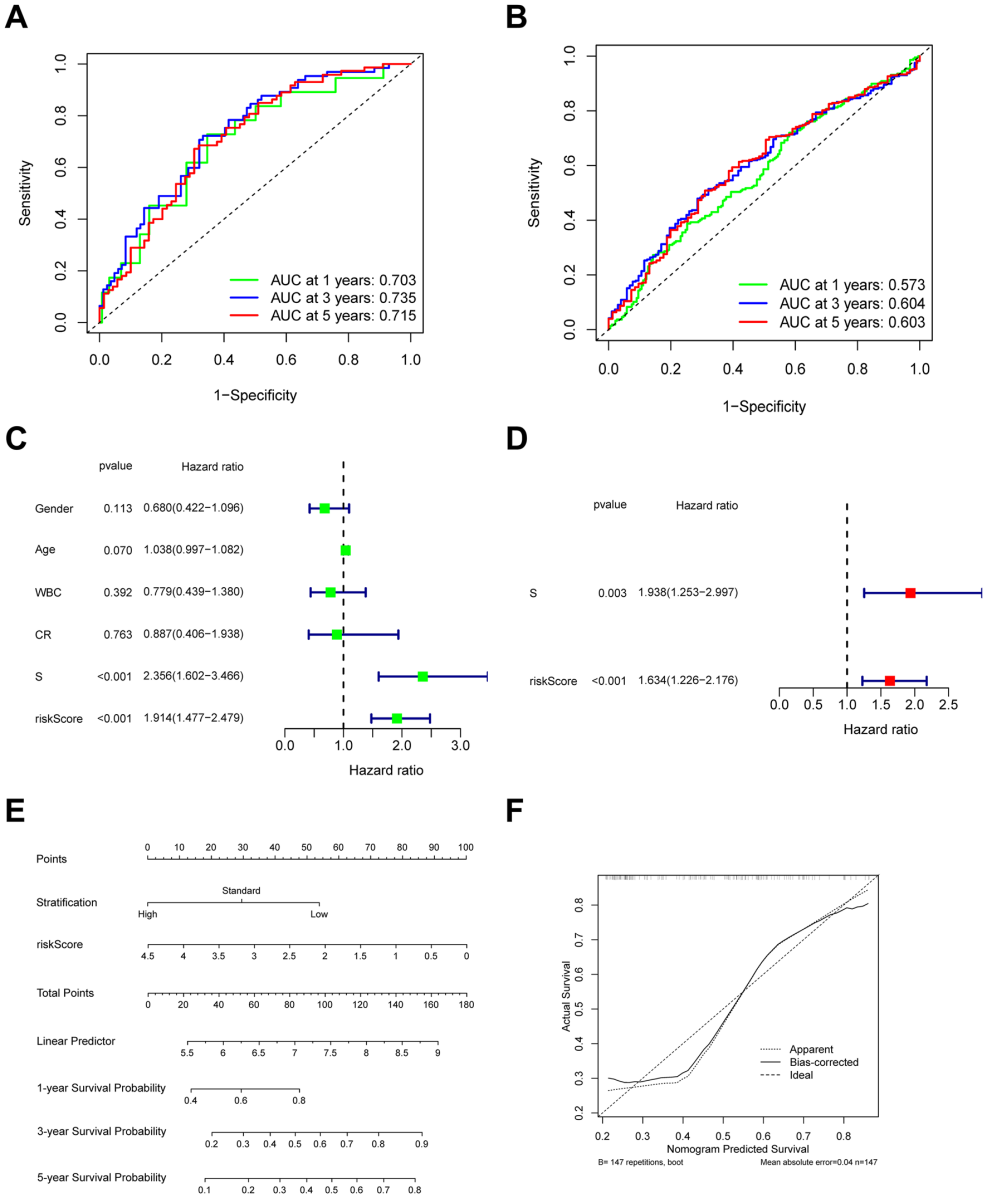
Efficiency of the hypoxia risk signature in prognostic prediction in AML. (**A**, **B**) ROC curves which showed the hypoxia risk signature’s predictive efficiency on the survival rate in AML from TARGET and GEO databases; (**C**, **D**) Univariate and multivariate Cox analyses which evaluated the risk signature’s independence in prognostic value in terms of overall survival in pediatric AML patients; (**E**) Nomograms for the probability of death at 1-, 3- and 5-years; (**F**) The calibration curve of the nomograms.

To evaluate the efficiency of the hypoxia risk model in predicting the 1-, 3-, and 5-years survival rates, we used data obtained from the TARGET and GEO datasets to carry out a received operating characteristic (ROC) curve. The area under the ROC curve (AUC) was respectively 0.703, 0.735 and 0.715 at 1, 3, and 5-years, suggesting the hypoxia risk model had a high prognostic value (Fig. 4A). Data from GEO dataset further confirmed the predictive value of the hypoxia risk signature (Fig. 4B).

Patients with higher scores seemed to have a higher likelihood of developing hypoxia in the tumor immune microenvironment. Cut points based on the Youden Index were used to serve as the standard for the classification of the risk scores. The cut-off value divided AML patients into a high-risk group and a low-risk group. Patients that were classified as high risk had a significantly lower chance of surviving. The independent efficiency in prognosis prediction of the risk signature in terms of OS in TARGET was then evaluated using univariate and multivariate Cox tests by combining the clinical characteristics of pediatric AML patients. We used “WBC” to represent “white blood cell count at diagnosis”, and “CR” to represent “complete remission status at end of course 1”, both of which were main clinical indicators in evaluation of status and outcome of AML patients^24^. According to the univariate study, higher hypoxia risk levels were associated with lower survival chances (Fig. 4C). Risk stratification was another element linked to poor survival. Multivariate analyses further indicated that the hypoxia risk score was independently correlated with OS in pediatric AML patients (Fig. 4D), meaning that the risk score may be used as an independent prognostic indicator for AML. Using multivariable logistic regression analysis, a nomogram was developed (Fig. 4E). The corresponding calibration curve for the nomogram proved its precision, as seen in Fig. 4F.

### Identification of hypoxia-related signaling pathways with GSEA

**Figure 5:**
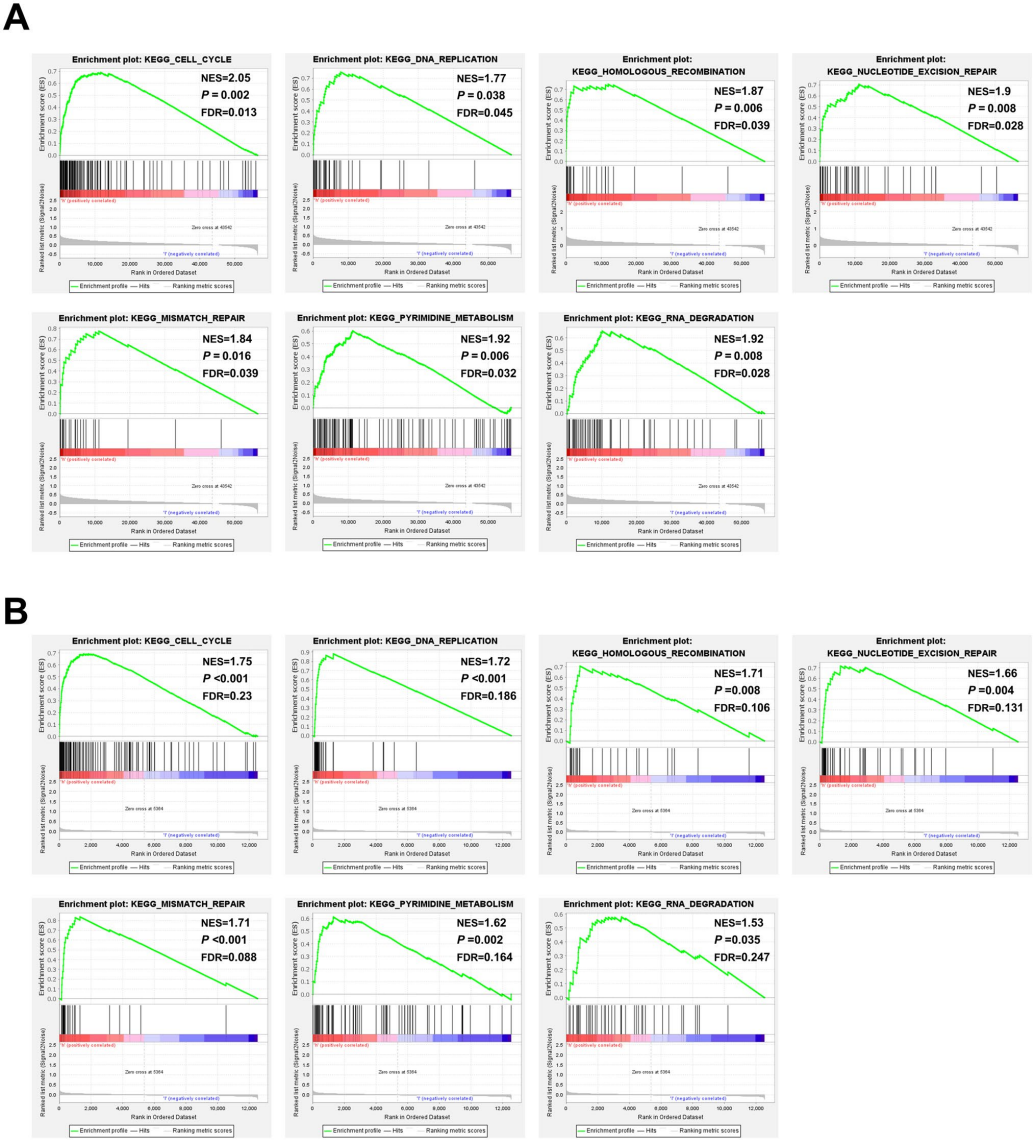
Identification of hypoxia-related signaling pathways with GSEA. (**A**) GSEA (http://www.gsea-msigdb.org/gsea/index.jsp) results of the gene KEGG enrichment in the AML patients from TARGET; (**B**) further validation by the GEO data.

We used GSEA to compare data of the two hypoxia risk groups to confirm related signaling mechanisms that were triggered in the high-risk group. In TARGET, gene sets, differentially enriched in AML patients with higher risk, were found related to processes which promoted anti-apoptosis, as well as cell proliferation, such as cell cycle, DNA replication, homologous recombination, nucleotide excision repair, mismatch repair, pyrimidine metabolism and RNA degradation (Fig. 5A). These results were double-checked in the GEO database, as shown in Fig. 5B. Besides, as shown in Supplementary Fig. 4, hypoxia gene set was differentially enriched in the high-risk groups of the TARGET (Supplementary Fig. 4A) and GEO (Supplementary Fig. 4B) databases (*P* < 0.05).

### Immune landscape between high and low hypoxia risk groups of AML patients

**Figure 6:**
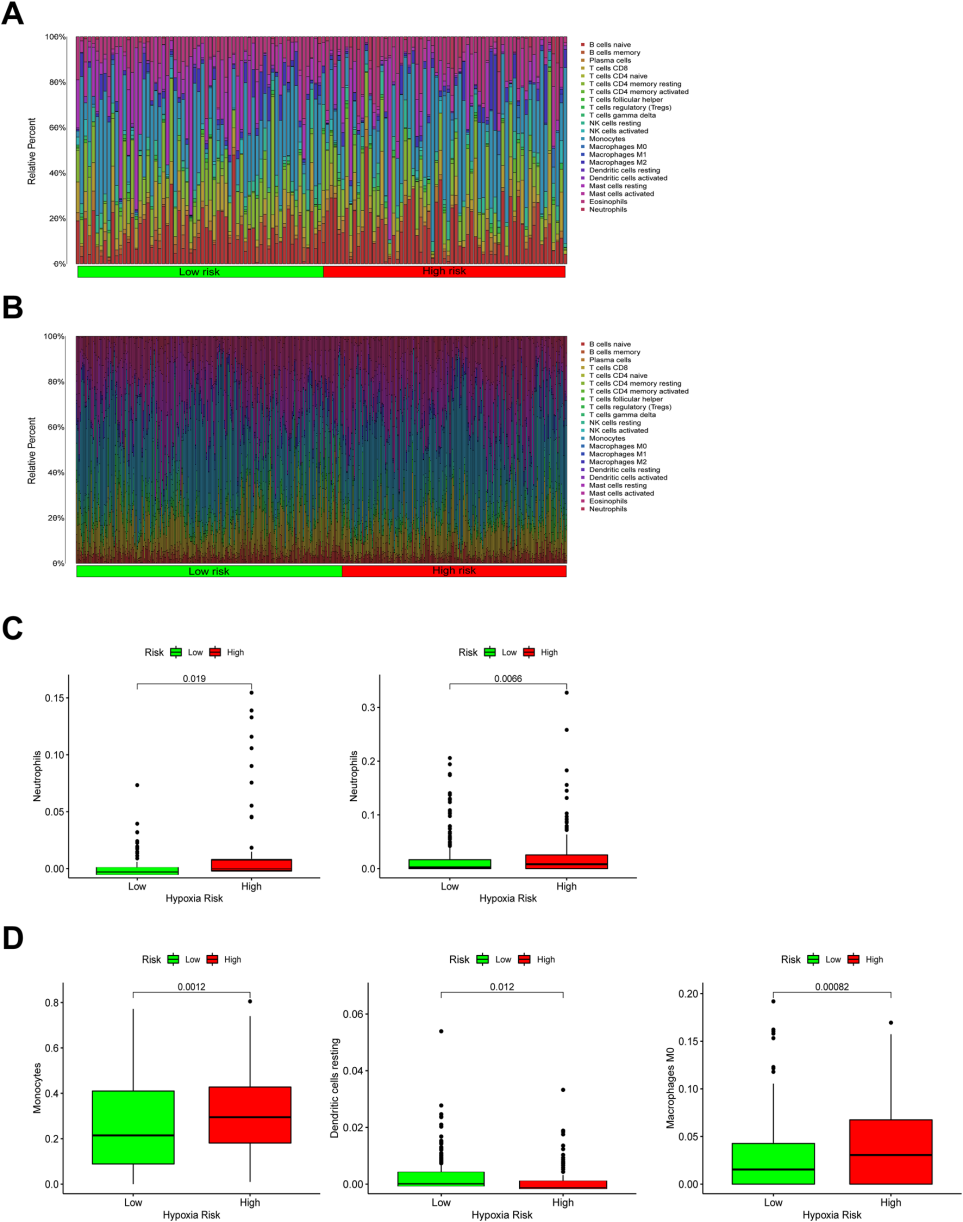
Immune landscape between high and low hypoxia risk groups of AML patients. (**A**, **B**) Relative proportion of immune infiltration of 22 immune cell types in two hypoxia risk groups in TARGET and GEO; (**C**, **D**) Box plots showing significantly differences of immune cells between groups with different hypoxia risk scores.

More and more evidence has shown that the hypoxic microenvironment could protect tumor cells from anti-tumor immune responses, for it might inhibit the anti-tumor function of immune effector cells, so that promote immune escape^25,26^. The capability of the established hypoxia risk signature in the immune microenvironment evaluation needed further explore. Hence, we estimated the differences in the 22 immune cell types’ immune penetration between low- and high-risk AML patients using the CIBERSORT method combining with the downloaded LM22 signature matrix. The results of AML patients from TARGET and GEO databases were summarized in Fig. 6A,B. In both TARGET and GEO, AML patients with low risk of hypoxia had significantly lower proportions of neutrophils, which have been defined as immunosuppressive cells linked to hypoxia (Fig. 6C). Furthermore, other immunosuppressive cells (such as TAM and monocytes) were shown to be higher in patients with high hypoxia risk than in patients with low hypoxia risk, while resting dendritic cells displayed the opposite proportion (Fig. 6D). Hence, focusing on hypoxia might have important therapeutic consequences in terms of enhancing immunotherapy.

### Formation of immunosuppressive microenvironment in high hypoxia risk group

**Figure 7:**
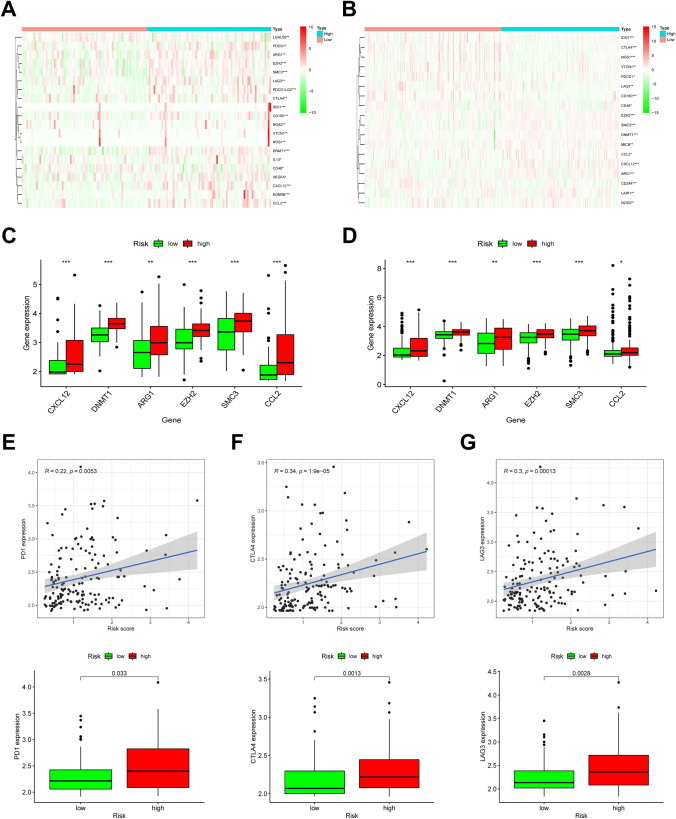
Formation of immunosuppressive microenvironment in high hypoxia risk group. (**A**, **B**) Heatmaps of the expression profiles of genes which negatively regulated the Cancer-Immunity Cycle in TARGET (https://ocg.cancer.gov/programs/target) and GEO (https://www.ncbi.nlm.nih.gov/geo); (**C**, **D**) The expression levels of immunosuppressive cytokines in two hypoxia risk groups; *P < 0.05, **P < 0.01 and ***P < 0.001; (**E**–**G**) Correlation between expressions of three immune checkpoints (PD1, CTLA-4, and LAG3) and hypoxia risk score, and their expressions in two hypoxia risk groups of AML patients.

Cancer immunotherapy studies in recent years are mostly based on the intellectual framework according to the Cancer-Immunity Cycle^27^. And the Cancer-Immunity Cycle actually refers to a series of events that eliminate cancer by affecting the immune system's ability^28,29^. These processes are often inhibited by several genes, whose expression levels are often increased in the immunosuppressive microenvironment of tumors^30^. In this study, we downloaded the gene signatures from the website Tracking Tumor Immunophenotype, and then explored the expression levels of genes with negative regulation in high and low hypoxia risk groups. Genes were found upregulated in the high hypoxia risk group, as shown in Fig. 7A, B, implying that patients with higher hypoxia risk had lower activities of those processes.

We further examined the expression levels of the immunosuppressive cytokines (Supplementary Table 3) and that of the identified immune checkpoints in the low and high hypoxia risk groups, considering previous indications that these molecules may be upregulated under hypoxic conditions. All immunosuppressive cytokines in Supplementary Table 3 were extracted from a signature set file, which was downloaded from an online website named Tracking Tumor Immunophenotype (TIP, http://biocc.hrbmu.edu.cn/TIP/index.jsp)^31^, which was a user-friendly one-stop shop web tool developed to comprehensively resolve tumor immunophenotype. The signature set file contained 23 confirmed signature sets involved in seven-step anticancer immunity^32,33^. After exploring the expression levels of all the immunosuppressive cytokines between two risk groups, we found that the expression levels of the six ones (ARG1, CCL2, CXCL12, DNMT1, EZH2 and SMC3) in Fig. 7C & D, were significantly higher in the high-risk groups than those in low-risk groups. PD1, which was positively associated with hypoxia risk score, was also upregulated in AML patients with higher hypoxia risk (Fig. 7E). Moreover, the expression levels of another two essential immune checkpoints (CTLA-4 and LAG-3) in the high hypoxia risk group were meaningfully higher than those in the low hypoxia risk group (Fig. 7F, G).

These findings suggested that patients with higher hypoxia risk scores established an immunosuppressive microenvironment as a consequence of upregulated immunosuppressive cytokines and immune checkpoints.

## Discussion

Significant evidence suggests that tumor hypoxia plays a role in those processes that give tumor cells a growth advantage and lead to the formation of malignant phenotypes^3^. Hypoxia in tumors has been proposed to serve as a prognostic indicator for patient outcome^34^. While techniques such as biomarkers' expressions through immunohistochemistry have been used to detect the degree of hypoxia in tumors, a definitive method is still unknown and urgently needed^35,36^.

The risk model we established mainly consisted of four genes associated with hypoxia, and most of them were found upregulated in hypoxia. The four identified genes were also found respectively correlated with poor OS in childhood AML. It has been reported that ENO3, an important glycolysis related gene, also participates in HIF-1 signaling pathway, which is essential in response to hypoxia^37^. Also, the activity of ENO3 were found upregulated in tumor cells, and affected their biological function^38^. As a key factor in cell cycle, CCNA2 could be up-regulated upon hypoxia-induced activation to promote cell proliferation and tumorigenesis^39,40^, and has been identified as tumor therapeutic target^41^. In addition, F3 is confirmed related with hypoxia-associated responses^42^. SLC2A5 promotes cell growth and metastasis in lung cancer, where hypoxia is generally associated with disease progression and poor prognosis^43^.

There have been several risk models composed of multiple genes in clinic that could predict the clinical prognosis of patients who suffer from tumors. Here, the risk model we developed to serve as a convenient detection in clinic, consisted of four genes associated with hypoxia. Figures 3 and 4 revealed that the risk signature we developed may be used as an independent prognostic indicator for AML patients from TARGET. Due to the lack of clinical information on AML samples in GEO, we cannot group AML patients in GEO according to the clinical risk stratification to further analyze the relationships between the clinical risk stratification of AML patients and the expression levels of SLC2A5, F3, ENO3 or CCNA2, just like that in the TARGET (Fig. 3B). The lack of information in the GEO data set is also one of the limitations of this manuscript. We will collect more clinical sample data in subsequent experimental studies for further verification.

The GSEA analyses showed that in TARGET and GEO, the enriched pathways that gene sets in AML patients with higher risk mainly included cell cycle, DNA replication, homologous recombination, nucleotide excision repair, mismatch repair, pyrimidine metabolism and RNA degradation (Fig. 5), as well as hypoxia (Supplementary Fig. 4). Cell cycle can alter the hypoxia response^44^. High degree of hypoxia can induce cell cycle arrest in a variety of cell types^45^, while CCNA2 is a key factor in cell cycle and could be up-regulated induced by hypoxia to promote cell proliferation^39^. Also, acute stress caused by hypoxia arrests DNA replication and triggers DNA damage^46^, while DNA replication supported by the complexes of CCNA2‐CDK could push forward the cell cycle in the S‐phase^47^. Besides, hypoxia has been reported to suppress DNA repair through homologous recombination^48^, while CCNA2 is located at sites of DNA double-strand breaks (DSB) and plays a role in homologous recombination^49^. In addition, nucleotide excision repair^50^, mismatch repair^51^, pyrimidine metabolism^52^ and RNA degradation^53^ have been reported associated with hypoxia. Although these four pathways have not been reported directly link to the four genes by now, all the gene sets differentially enriched in the high-risk groups were closely associated with hypoxia.

Increasing evidence shows that hypoxia promotes the function of suppressive cells (such as, neutrophils and TAMs) and the release of immunosuppressive molecular to protect tumor cells away from natural immune responses^54^. In our study, neutrophils, a kind of the main immunosuppressive cells, were indeed increased in the AML patients in the high hypoxia risk group in TARGET and GEO. The activation of tumor antigen-specific dendritic cells is one of the most important steps in the anti-tumor immune surveillance process. Some studies have reported that hypoxia could inhibit the growth and activation of dendritic cells. For example, low oxygen levels impaired the maturation and function of dendritic cells for hypoxia upregulated microRNA 21, which further down-regulated the expression of CD80, CD86 and MHCII^55^. Tran et al. pointed out that hypoxia-inducible factor 1 (HIF-1) limits dendritic cell stimulation of CD8 + T cell immunity^56^. In line with this evidence, our results showed that resting dendritic cells were higher in pediatric AML patients in the high hypoxia risk group, suggesting these patients were in an immune disability status.

Macrophages in the tissues of tumors are designated as TAMs, and they were mainly classified into M1-like macrophages and M2-like macrophages, both of which are polarized from MΦ macrophages. Previous studies support that MΦ macrophages migrate at the tumor site only when hypoxia takes place. The M2 macrophages dominates in hypoxic niches, since hypoxia is the major trigger for the transition from MΦ to the M2 phenotype^57^. Hence, hypoxia is considered playing an essential role in the regulation of TAMs phenotype transformation, for that factors released by the hypoxic TAMs can help promote tumor growth, and cancer immunosuppression^58,59^. Results of CIBERSORT analysis showed that pediatric AML patients with higher hypoxia risk scores had significantly higher proportions of MΦ macrophages. Furthermore, it is well known that monocytes from the peripheral blood are drawn to tumor lesions, where they undergo terminal differentiation into macrophages. Consistent with this evidence, monocytes in the high hypoxia risk group were found increased, and were significantly higher than those in the low hypoxia risk group, further confirming the predictive value of the risk signature in immune microenvironment of AML.

Cytokines are molecules that play a crucial role in immune responses to tumors. Immune cell exhaustion is mostly caused by tumor immunosuppressive cytokines. As CXCL12 promoter contains two HIF-1α binding sites, several studies have pointed out that hypoxia in tumors could upregulate the expression of CXCL12 and induce its secretion^60,61^, which in turn inhibited the function of the immune system by preventing cytotoxic T cells from infiltrating the tumor and killing cancer cells^62,63^. ARG1, a crucial immunosuppressive mediator, has been shown to boost immune evasion mediated by myeloid cells and promote tumor growth^64^. EZH2 is a key regulator of AML^65^, and the inhibition of EZH2 has been demonstrated to reduce tumor growth and promote apoptosis by regulating T cell polyfunctionality and survival^66,67^. In this hypoxia-associated study, immunosuppressive cytokines were found to be significantly upregulated in high hypoxia risk AML patients, which further promoted the suppression of immune.

Moreover, immune checkpoints are essential in carcinogenesis for its effects of promoting immunosuppression in tumors. Tumor cells stimulate those immune checkpoint targets to prevent themselves away from the attack by immune cells. At present, the common targets of immune checkpoints mainly include PD1, PD-L1, CTLA-4, LAG3, TIM-3, and TIGIT. Hypoxia has been shown to induce PD-1 and CTLA-4 selectively upregulated in the tumor microenvironment^68–70^. Additionally, repressing hypoxia could lead to the decreased expression of LAG3 in colon cancer^71^. Here, immune checkpoints (PD1, CTLA-4, and LAG3) were meaningfully upregulated in the pediatric AML patients with high hypoxia risk scores.

This was the first research of its kind to create a hypoxia risk model composed of four genes for childhood AML. This risk signature, which was also applied to adult AML, could be used as an independent prognostic indicator for pediatric AML patients in clinic. It reflected the overall intensity of the immune response in the AML microenvironment. As a result of our findings, we now have a better idea of how hypoxia influences the clinical prognosis and the immune microenvironment in AML, which could help develop potential therapies targeting hypoxia.

## Methods

### Datasets

The RNA-seq transcriptome details of pediatric AML patients, as well as the corresponding clinical information, were extracted from Therapeutically Applicable Research to Generate Effective Treatments (TARGET) database (https://ocg.cancer.gov/programs/target) as a training set. A testing set for validation mainly includes all information of AML patients downloaded from Gene Expression Omnibus (GEO) online database (https://www.ncbi.nlm.nih.gov/geo/query/acc.cgi?acc=GSE37642)72. The corresponding information was shown in Supplementary Tables 4, 5. Information of AML patients downloaded from The Cancer Genome Atlas (TCGA) database (https://www.cancer.gov/tcga) was also used as an external validation. The heat maps based on the data of these databases were generated by using R software (version 3.6.2), which was downloaded online (https://www.r-project.org/).

### Analysis of integration of protein–protein interaction (PPI) network

We built a network of protein–protein interaction (PPI) using the STRING database to explore the interaction relationships of the primary ones of those hypoxia-associated genes, which were downloaded from Gene Set Enrichment Analysis (hallmark-hypoxia). Then we calculated the numbers of interconnections using the Network Analyzer plug-in and labeled them as node degree. The hypoxia-related genes were ranked in descending order according to their node degree. The top 150 genes were chosen as the candidate hypoxia associated genes in childhood AML.

### Estimation of immune cell type fractions

An analytical tool, CIBERSORT, uses gene expression data to estimate the abundances of cell types in a mixed cell population. A leukocyte gene signature matrix known as LM22 in CIBERSORT, which contains 547 genes, was utilized to identify 22 types of immune cells^73^, which mainly includes tumor-associated macrophages (TAMs), resting dendritic cells, neutrophils and so on. The fractions of immune cell types between two groups with different hypoxia risk scores were estimated using CIBERSORT.

### Survival analysis and development of a risk model

The survival package and the survminer package for Kaplan–Meier analyses were used to compare overall survival (OS) between the two hypoxia risk groups, while the results of Kaplan–Meier analyses in Supplementary Fig. 1 were generated by a specific online website for pediatric cancers (http://pedtranscriptome.org/). Then, we used univariable Cox regression analysis to identify the genes related with hypoxia, which were significantly associated with OS and might serve as candidate prognostic indicators in AML. Next, multivariate Cox analysis was carried out to further achieve the coefficients of the identified genes. The formula of the risk score was next constructed as followed: risk score = $\sum_{\text{i=1}}^{{\rm n}}{\text{Coe}}{(}{\text{i}}{) }\times \text{Exp}{(}{\text{i}}{)}$. In this risk score, n meant four, while the Coe(i) represented the multivariable Cox regression coefficients of the four identified hypoxia genes, and Exp(i) was their corresponding expression level. After calculating the risk score of each AML sample according to the risk model, we chose the median value as the cutoff, based on which the AML samples were divided into the high-risk group and the low-risk group. The information of two hypoxia risk groups in two databases were shown in Supplementary Table 6. To verify the accuracy and effectiveness of the developed risk model in predicting OS of AML patients, ROC curves were generated.

### Data of gene set enrichment analysis (GSEA)

We identified the meaningful difference of gene set between two groups with different hypoxia risks in the enrichment of the Molecular Signatures Database (MSigDB) collection (h.all.v7.4.symbols.gmt) using GSEA^74,75^, which was downloaded online (http://www.gsea-msigdb.org/gsea/index.jsp). For each analysis, the permutations of gene set were all performed 1,000 times. The KEGG pathways enrichment analyses were also done by using the GSEA software^75,76^. Besides, the phenotype label was then used to serve as a risk score.

### Consent for publication

All listed authors took part actively in the research and read and approved the manuscript submitted.

## Supplementary Information

Supplementary Table 1.

Supplementary Table 2.

Supplementary Table 3.

Supplementary Table 4.

Supplementary Table 5.

Supplementary Table 6.

Supplementary Figures.

Supplementary Figure 1.

Supplementary Figure 2.

Supplementary Figure 3.

Supplementary Figure 4.

## Data Availability

The generated and analyzed datasets of this research are available in TARGET (https://ocg.cancer.gov/programs/target), GEO (https://www.ncbi.nlm.nih.gov/geo) and TCGA (https://www.cancer.gov/tcga) both of which are freely available to the public.

## References

[1] Petrova V, Annicchiarico-Petruzzelli M, Melino G, Amelio I (2018). The hypoxic tumour microenvironment. Oncogenesis.

[2] Muz B, de la Puente P, Azab F, Azab AK (2015). The role of hypoxia in cancer progression, angiogenesis, metastasis, and resistance to therapy. Hypoxia (Auckl).

[3] Jing X (2019). Role of hypoxia in cancer therapy by regulating the tumor microenvironment. Mol. Cancer..

[4] Vujkovic M (2017). Genomic architecture and treatment outcome in pediatric acute myeloid leukemia: A children's oncology group report. Blood.

[5] Pogosova-Agadjanyan EL (2020). Aml risk stratification models utilizing Eln-2017 guidelines and additional prognostic factors: A swog report. Biomark Res..

[6] Edwards H (2009). Runx1 regulates phosphoinositide 3-kinase/akt pathway: Role in chemotherapy sensitivity in acute megakaryocytic leukemia. Blood.

[7] Wang Y (2014). Echinomycin protects mice against relapsed acute myeloid leukemia without adverse effect on hematopoietic stem cells. Blood.

[8] Kremer KN (2013). Cxcr4 chemokine receptor signaling induces apoptosis in acute myeloid leukemia cells via regulation of the Bcl-2 family members Bcl-Xl, Noxa, and Bak. J. Biol. Chem..

[9] Bernasconi P, Farina M, Boni M, Dambruoso I, Calvello C (2016). Therapeutically targeting self-reinforcing leukemic niches in acute myeloid leukemia: A worthy endeavor?. Am. J. Hematol..

[10] Velasco-Hernandez T (2019). Hif-1Α deletion may lead to adverse treatment effect in a mouse model of Mll-Af9-driven aml. Stem Cell Reports..

[11] Griessinger E (2014). A niche-like culture system allowing the maintenance of primary human acute myeloid leukemia-initiating cells: A new tool to decipher their chemoresistance and self-renewal mechanisms. Stem Cells Transl. Med..

[12] Cubillos-Zapata, C. et al. Hypoxia-induced Pd-L1/Pd-1 crosstalk impairs T-cell function in sleep apnoea. *Eur. Respir. J.* **50**, (2017). 10.1183/13993003.00833-2017 29051270

[13] Chambers AM, Matosevic S (2019). Immunometabolic dysfunction of natural killer cells mediated by the hypoxia-Cd73 axis in solid tumors. Front. Mol. Biosci..

[14] Maimela NR, Liu S, Zhang Y (2019). Fates of Cd8+ T cells in tumor microenvironment. Comput. Struct. Biotechnol. J..

[15] Terry S, Buart S, Chouaib S (2017). Hypoxic stress-induced tumor and immune plasticity, suppression, and impact on tumor heterogeneity. Front. Immunol..

[16] Duffy MJ, Crown J (2019). Biomarkers for predicting response to immunotherapy with immune checkpoint inhibitors in cancer patients. Clin. Chem..

[17] Qi L, Chen J, Yang Y, Hu W (2020). Hypoxia correlates with poor survival and M2 macrophage infiltration in colorectal cancer. Front Oncol..

[18] Zhang X (2020). Sohlh2 alleviates malignancy of eoc cells under hypoxia via inhibiting the Hif1Α/Ca9 signaling pathway. Biol. Chem..

[19] Pogosova-Agadjanyan EL (2020). Aml risk stratification models utilizing Eln-2017 guidelines and additional prognostic factors: A swog report. Biomark. Res..

[20] Herold T (2020). Validation and refinement of the revised 2017 European leukemianet genetic risk stratification of acute myeloid leukemia. Leukemia.

[21] Feng J (2020). Hypoxia-induced circccdc66 promotes the tumorigenesis of colorectal cancer via the mir-3140/autophagy pathway. Int. J. Mol. Med..

[22] Li J (2018). Comprehensive analysis of differentially expressed non-coding rnas and mrnas in gastric cancer cells under hypoxic conditions. Am. J. Transl. Res..

[23] Greenhough, A. et al. Cancer cell adaptation to hypoxia involves a hif-Gprc5a-yap axis. *Embo Mol. Med.* **10**, e8699 (2018). 10.15252/emmm.201708699 PMC6220329 30143543

[24] Scandura JM (2011). Phase 1 study of epigenetic priming with decitabine prior to standard induction chemotherapy for patients with aml. Blood.

[25] Noman MZ (2015). Hypoxia: A key player in antitumor immune response. A review in the theme: Cellular responses to hypoxia. Am. J. Physiol. Cell Physiol..

[26] Vaupel P, Multhoff G (2017). Accomplices of the hypoxic tumor microenvironment compromising antitumor immunity: Adenosine, lactate, acidosis, vascular endothelial growth factor, potassium ions, and phosphatidylserine. Front. Immunol..

[27] Chen DS, Mellman I (2013). Oncology meets immunology: The cancer-immunity cycle. Immunity.

[28] Ok CY, Young KH (2017). Targeting the programmed death-1 pathway in lymphoid neoplasms. Cancer Treat. Rev..

[29] Pio R, Ajona D, Ortiz-Espinosa S, Mantovani A, Lambris JD (2019). Complementing the cancer-immunity cycle. Front. Immunol..

[30] Yasukawa M (2011). Guest editorial: Cancer immunology. Int. J. Hematol..

[31] Xu L (2018). Tip: A web server for resolving tumor immunophenotype profiling. Cancer Res..

[32] Harlin H (2009). Chemokine expression in melanoma metastases associated with Cd8+ T-cell recruitment. Cancer Res..

[33] Nagarsheth N, Wicha MS, Zou W (2017). Chemokines in the cancer microenvironment and their relevance in cancer immunotherapy. Nat. Rev. Immunol..

[34] Jubb AM, Buffa FM, Harris AL (2010). Assessment of tumour hypoxia for prediction of response to therapy and cancer prognosis. J. Cell. Mol. Med..

[35] Walsh JC (2014). The clinical importance of assessing tumor hypoxia: Relationship of tumor hypoxia to prognosis and therapeutic opportunities. Antioxid. Redox Signal..

[36] Koch CJ, Evans SM (2015). Optimizing hypoxia detection and treatment strategies. Semin. Nucl. Med..

[37] Mzoughi S (2020). Prdm15 is a key regulator of metabolism critical to sustain B-cell lymphomagenesis. Nat. Commun..

[38] Xie H (2017). Proteomics analysis to reveal biological pathways and predictive proteins in the survival of high-grade serous ovarian cancer. Sci. Rep..

[39] Zhang L (2020). Blockade of Jak2 protects mice against hypoxia-induced pulmonary arterial hypertension by repressing pulmonary arterial smooth muscle cell proliferation. Cell Prolif..

[40] Bai H (2019). Lncrna expression reveals the potential regulatory roles in hepatocyte proliferation during rat liver regeneration. Biomed. Res. Int..

[41] Hao M (2020). Identification of hub genes and small molecule therapeutic drugs related to breast cancer with comprehensive bioinformatics analysis. PeerJ.

[42] Grigoryev DN (2007). Exon-based mapping of microarray probes: Recovering differential gene expression signal in underpowered hypoxia experiment. Mol. Cell Probes..

[43] Lin YJ (2017). Tumor hypoxia regulates forkhead box C1 to promote lung cancer progression. Theranostics.

[44] Druker J (2021). Role of hypoxia in the control of the cell cycle. Int. J. Mol. Sci..

[45] Wang Z (2018). Baicalin induces cellular senescence in human colon cancer cells via upregulation of depp and the activation of Ras/Raf/Mek/Erk signaling. Cell Death Dis..

[46] Prasad D, Arora D, Nandicoori VK, Muniyappa K (2019). Elucidating the functional role of mycobacterium smegmatis recx in stress response. Sci. Rep..

[47] Li X (2019). Downregulation of Ccna2 disturbs trophoblast migration, proliferation, and apoptosis during the pathogenesis of recurrent miscarriage. Am. J. Reprod. Immunol..

[48] Yao T (2020). Aldh-1-positive cells exhibited a radioresistant phenotype that was enhanced with hypoxia in cervical cancer. BMC Cancer.

[49] Gygli PE (2016). Cyclin a2 promotes dna repair in the brain during both development and aging. Aging (Albany NY).

[50] Rezvani HR (2010). Hypoxia-inducible factor-1alpha regulates the expression of nucleotide excision repair proteins in keratinocytes. Nucleic Acids Res..

[51] Rodríguez-Jiménez FJ, Moreno-Manzano V, Lucas-Dominguez R, Sánchez-Puelles JM (2008). Hypoxia causes downregulation of mismatch repair system and genomic instability in stem cells. Stem Cells..

[52] Amellem O, Sandvik JA, Stokke T, Pettersen EO (1998). The retinoblastoma protein-associated cell cycle arrest in s-phase under moderate hypoxia is disrupted in cells expressing Hpv18 E7 oncoprotein. Br. J. Cancer.

[53] Shi Q (2016). Nitric oxide from brain microvascular endothelial cells may initiate the compensatory response to mild hypoxia of astrocytes in a hypoxia-inducible factor-1Α dependent manner. Am. J. Transl. Res..

[54] Lee CT, Mace T, Repasky EA (2010). Hypoxia-driven immunosuppression: A new reason to use thermal therapy in the treatment of cancer?. Int. J. Hyperthermia.

[55] Liang YR (2019). Interaction between bone marrow-derived dendritic cells and mir-21 of tubular renal epithelial cells under hypoxia. Eur. Rev. Med. Pharmacol. Sci..

[56] Tran CW (2020). Hypoxia-inducible factor 1 alpha limits dendritic cell stimulation of Cd8 T cell immunity. PLoS ONE.

[57] Leblond MM (2016). Hypoxia induces macrophage polarization and re-education toward an M2 phenotype in U87 and U251 glioblastoma models. Oncoimmunology.

[58] Murdoch C, Lewis CE (2005). Macrophage migration and gene expression in response to tumor hypoxia. Int. J. Cancer..

[59] Casazza A (2013). Impeding macrophage entry into hypoxic tumor areas by Sema3a/Nrp1 signaling blockade inhibits angiogenesis and restores antitumor immunity. Cancer Cell.

[60] Zhao K (2018). Lyg-202 inhibits activation of endothelial cells and angiogenesis through Cxcl12/Cxcr7 pathway in breast cancer. Carcinogenesis.

[61] Kim D (2014). Cxcl12 secreted from adipose tissue recruits macrophages and induces insulin resistance in mice. Diabetologia.

[62] Garg B (2018). Nfκb in pancreatic stellate cells reduces infiltration of tumors by cytotoxic T cells and killing of cancer cells, via up-regulation of Cxcl12. Gastroenterology.

[63] Spaan AN, Surewaard BG, Nijland R, van Strijp JA (2013). Neutrophils versus staphylococcus aureus: A biological tug of war. Annu. Rev. Microbiol..

[64] Steggerda SM (2017). Inhibition of arginase by Cb-1158 blocks myeloid cell-mediated immune suppression in the tumor microenvironment. J. Immunother. Cancer.

[65] Feng Y, Hu S, Li L, Peng X, Chen F (2020). Long noncoding Rna Hoxa-as2 functions as an oncogene by binding to Ezh2 and suppressing lats2 in acute myeloid leukemia (Aml). Cell Death Dis..

[66] Stacchiotti, S. et al. Comparative assessment of antitumor effects and autophagy induction as a resistance mechanism by cytotoxics and Ezh2 inhibition in ini1-negative epithelioid sarcoma patient-derived xenograft. *Cancers (Basel)*. **11**, 1015 (2019). 10.3390/cancers11071015 PMC6678245 31331120

[67] Zhao E (2016). Cancer mediates effector T cell dysfunction by targeting micrornas and Ezh2 via glycolysis restriction. Nat. Immunol..

[68] Noman MZ (2015). Tumor-promoting effects of myeloid-derived suppressor cells are potentiated by hypoxia-induced expression of mir-210. Cancer Res..

[69] Damgaci S (2018). Hypoxia and acidosis: Immune suppressors and therapeutic targets. Immunology.

[70] Gaber, T. et al. Ctla-4 mediates inhibitory function of mesenchymal stem/stromal cells. *Int. J. Mol. Sci.* **19**, 2312 (2018). 10.3390/ijms19082312 PMC6121442 30087255

[71] Yang Z (2020). Colon cancer combined with obesity indicates improved survival-research on relevant mechanism. Aging (Albany NY).

[72] Barrett T (2013). Ncbi Geo: Archive for functional genomics data sets-update. Nucleic Acids Res..

[73] Zeng Z, Xie D, Gong J (2020). Genome-wide identification of CPG island methylator phenotype related gene signature as a novel prognostic biomarker of gastric cancer. PeerJ.

[74] Mootha VK (2003). Pgc-1alpha-responsive genes involved in oxidative phosphorylation are coordinately downregulated in human diabetes. Nat. Genet..

[75] Subramanian A (2005). Gene set enrichment analysis: A knowledge-based approach for interpreting genome-wide expression profiles. Proc. Natl. Acad. Sci. USA.

[76] Kanehisa M, Furumichi M, Sato Y, Ishiguro-Watanabe M, Tanabe M (2021). Kegg: Integrating viruses and cellular organisms. Nucleic Acids Res..

