# Supplementary material for: A hypoxia risk signature for the tumor immune microenvironment evaluation and prognosis prediction in acute myeloid leukemia
Source: Sci Rep. 2021 Jul 19;11:14657. doi: 10.1038/s41598-021-94128-1 (PMC8289869; doi:10.1038/s41598-021-94128-1)
Supplement: Supplementary file 7 — Supplementary Figures. [file 41598_2021_94128_MOESM7_ESM.pdf]

Supplementary Figure Legends

Supplementary Figure 1

(A-D) Kaplan-Meier overall survival curves for patients assigned to high and low groups according to the expression levels of four identified genes, which were involved in the hypoxia risk signature. All of these figures were generated by a specific online website for pediatric cancers, named PDX for Childhood Cancer Therapeutics (PCAT, http://pedtranscriptome.org/).

Supplementary Figure 2

Kaplan-Meier overall survival curves for patients assigned to high and low groups according to the expression levels of HIF-1a in TARGET (A) and GEO (B).

Supplementary Figure 3

(A) Heatmap showing the expression profiles of the four identified hypoxia genes in groups with different hypoxia risks in TCGA (https://www.cancer.gov/tcga); (B) Distribution of patients' risk scores in the two risk groups from TCGA; (C) Distribution of patient status in the two hypoxia risk groups from TCGA; (D) Mortality rates of the two risk groups from TCGA; (E) Kaplan-Meier overall survival curves for AML patients in the two hypoxia risk groups from TCGA.

Supplementary Figure 4

Results of enrichment analysis using the hallmark gene sets database (h.all.v7.4.symbols.gmt) in GSEA in the AML patients from TARGET (A) and GEO (B).

Supplementary Table 1

Genes up-regulated in response to low oxygen levels.

Supplementary Table 2

The top 150 genes with higher associated node degrees.

Supplementary Table 3

The reported immunosuppressive cytokines.

Supplementary Table 4

The detail clinical characteristics of AML patients from TARGET database.

Supplementary Table 5

The detail clinical characteristics of AML patients from GEO database.

Supplementary Table 6

The original information of two hypoxia risk groups in TARGET and GEO.
