# Supplementary figures and images for: A hypoxia risk signature for the tumor immune microenvironment evaluation and prognosis prediction in acute myeloid leukemia
Source: Sci Rep. 2021 Jul 19;11:14657. doi: 10.1038/s41598-021-94128-1 (PMC8289869; doi:10.1038/s41598-021-94128-1)

A

ENO3

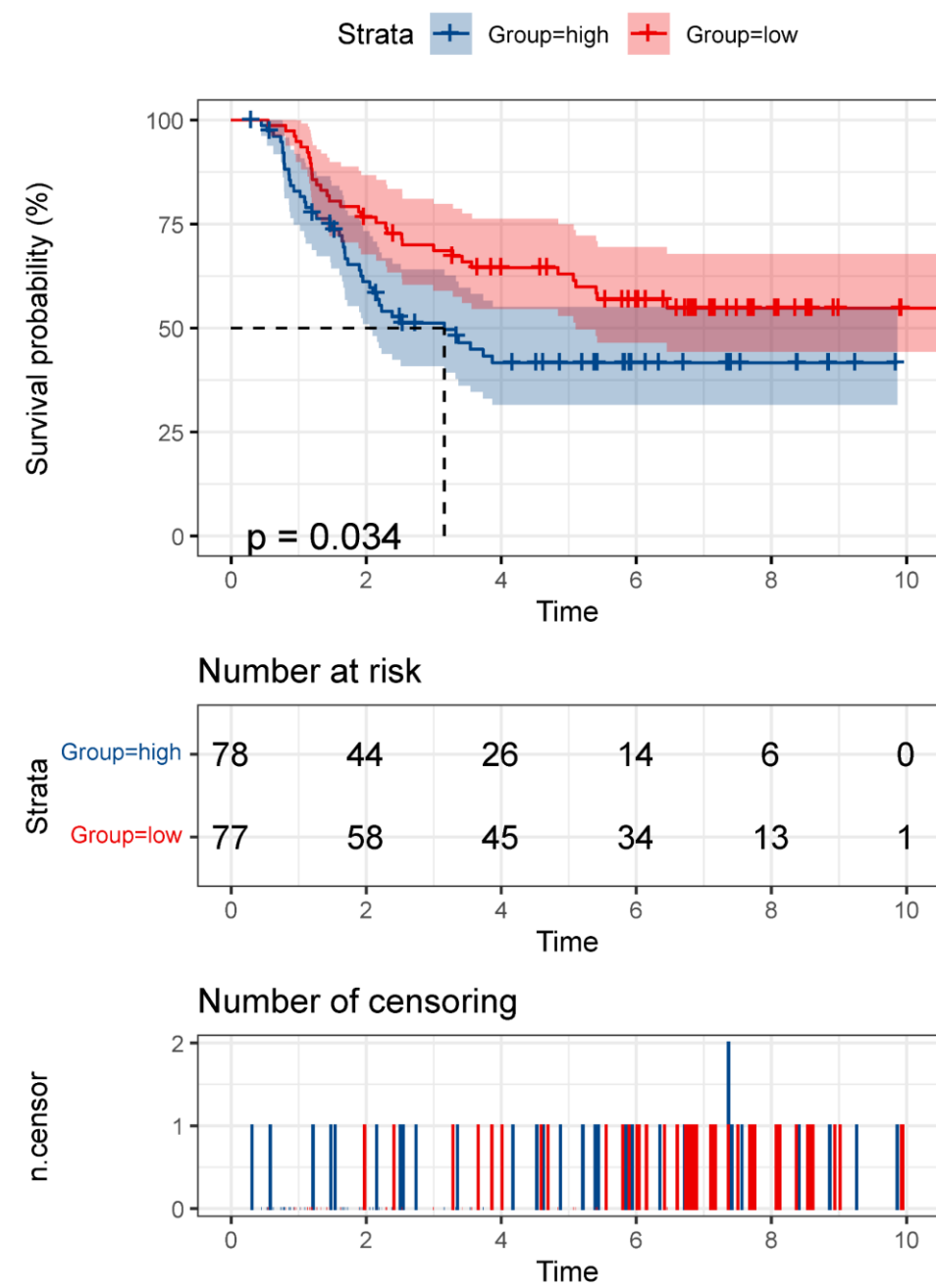

B

F3

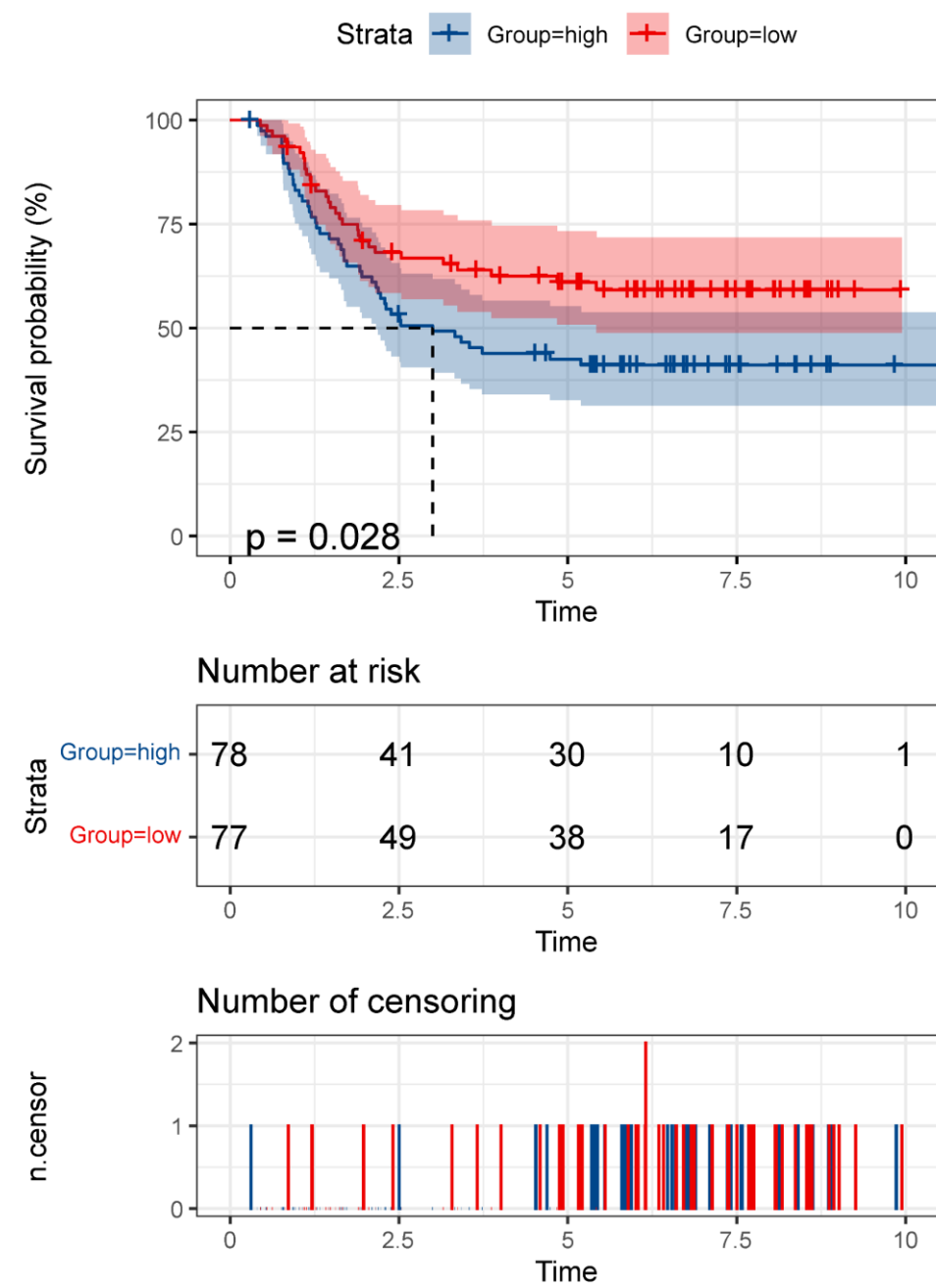

C

CCNA2

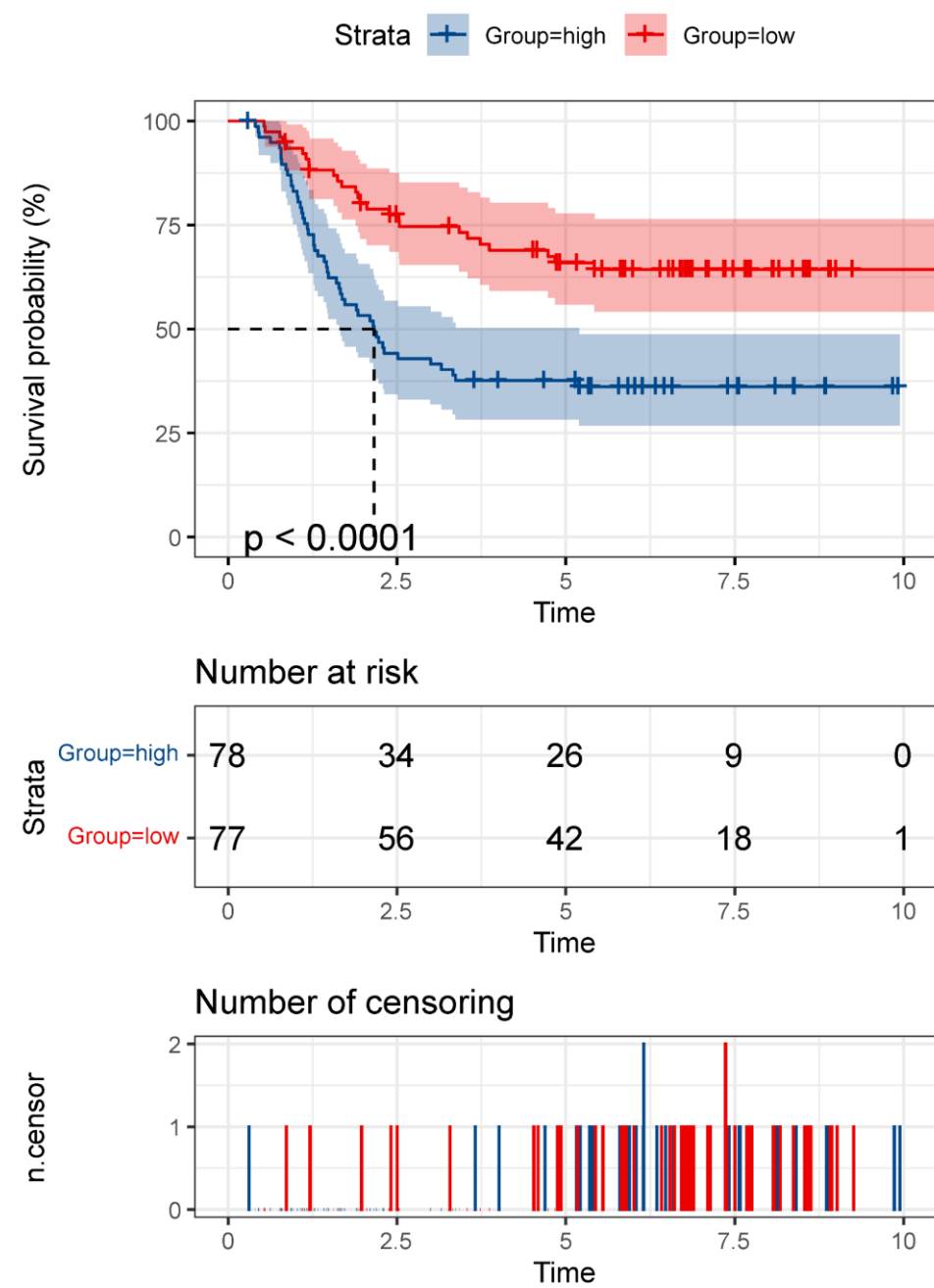

D

SLC2A5

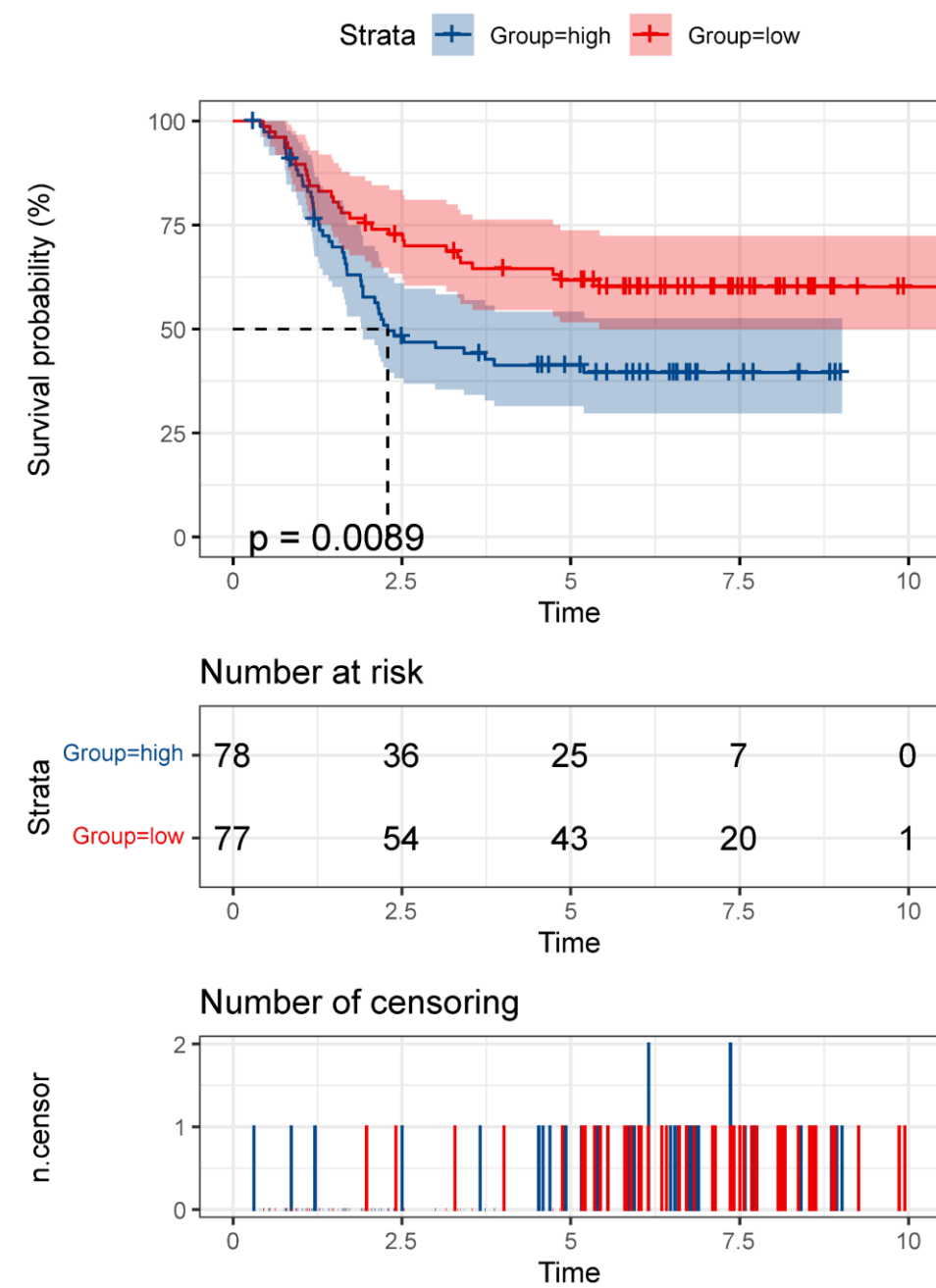

Supplement: Supplementary file 8 — Supplementary Figure 1. [file 41598_2021_94128_MOESM8_ESM.pdf]

**A**

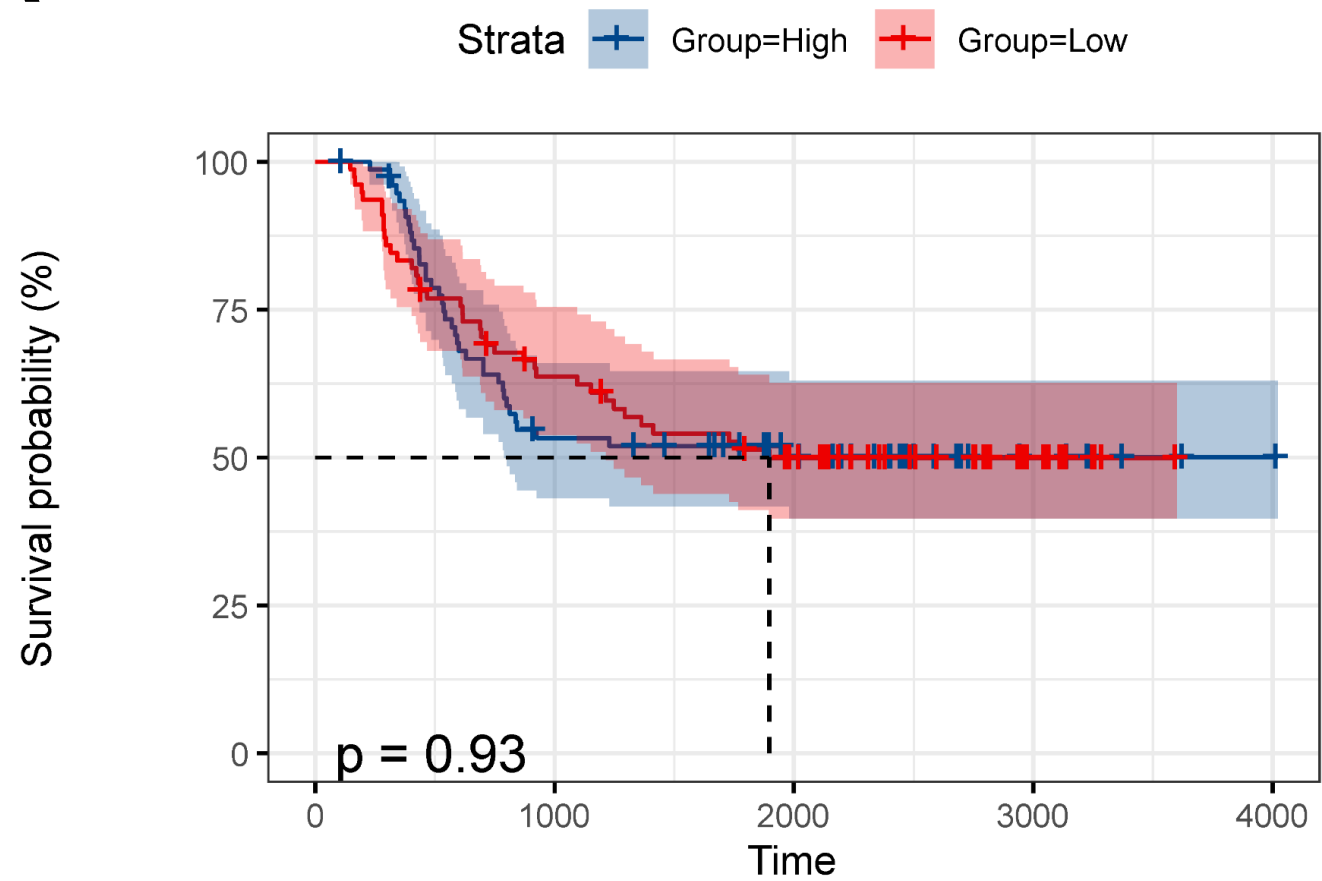

Number at risk

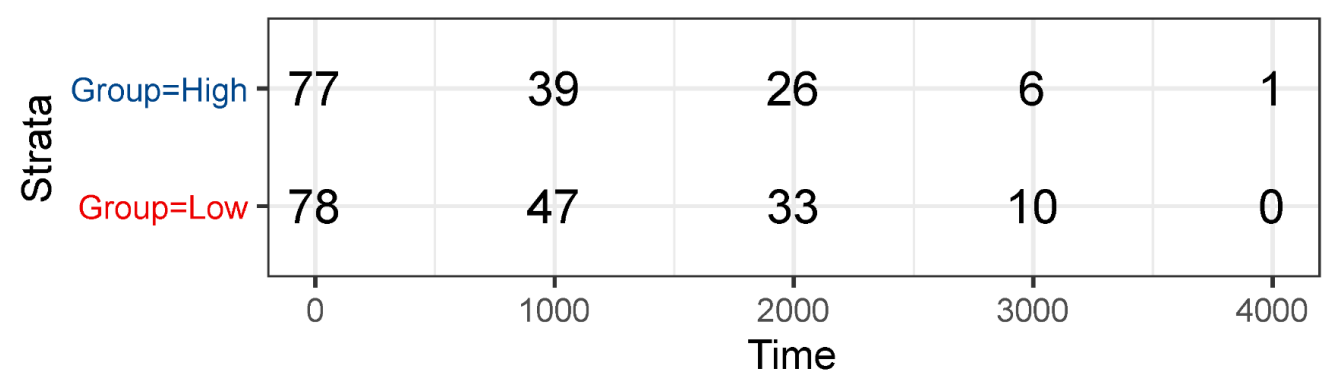

Number of censoring

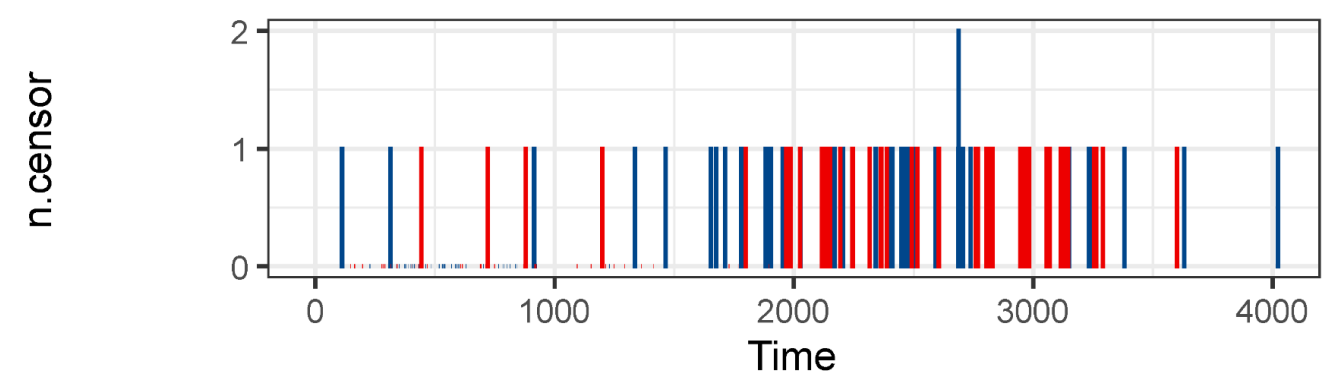

**B**

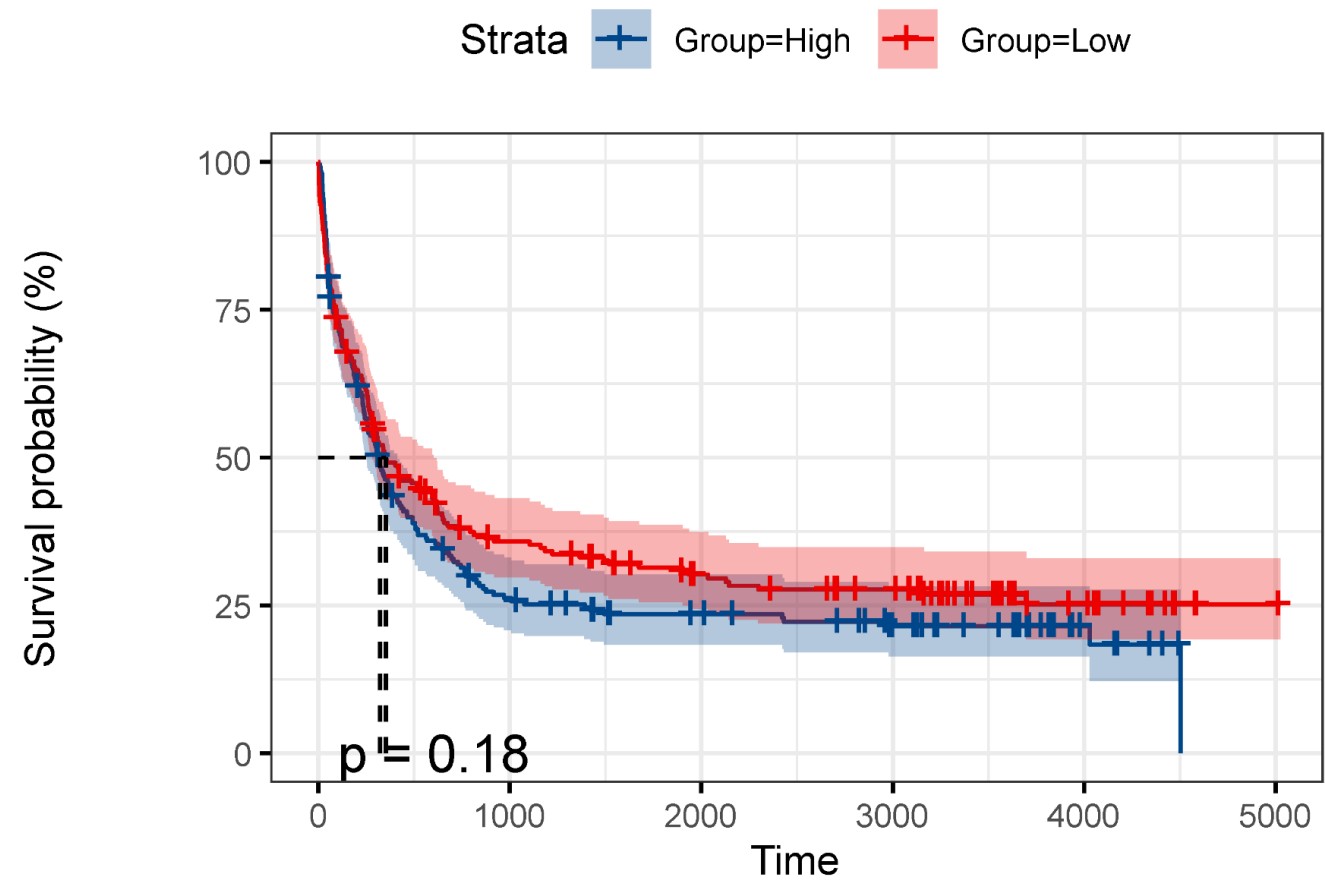

Number at risk

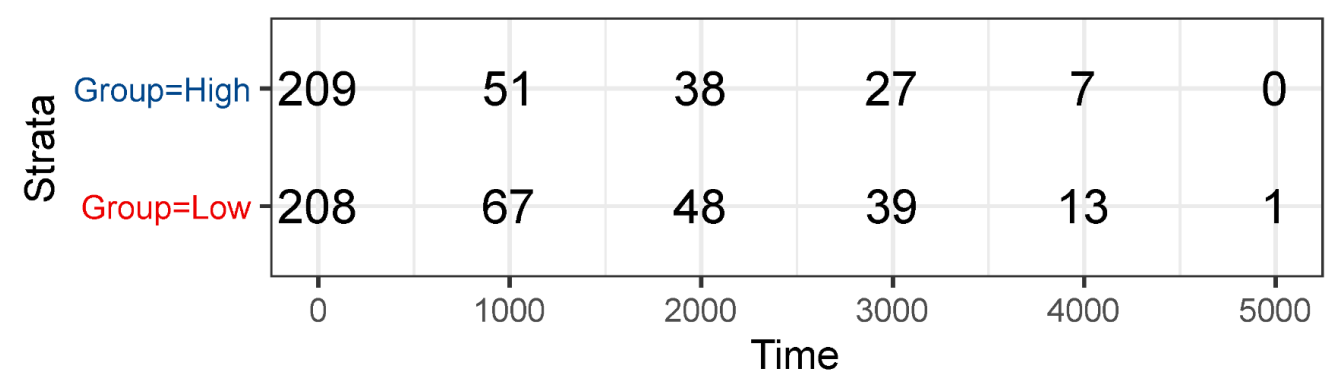

Number of censoring

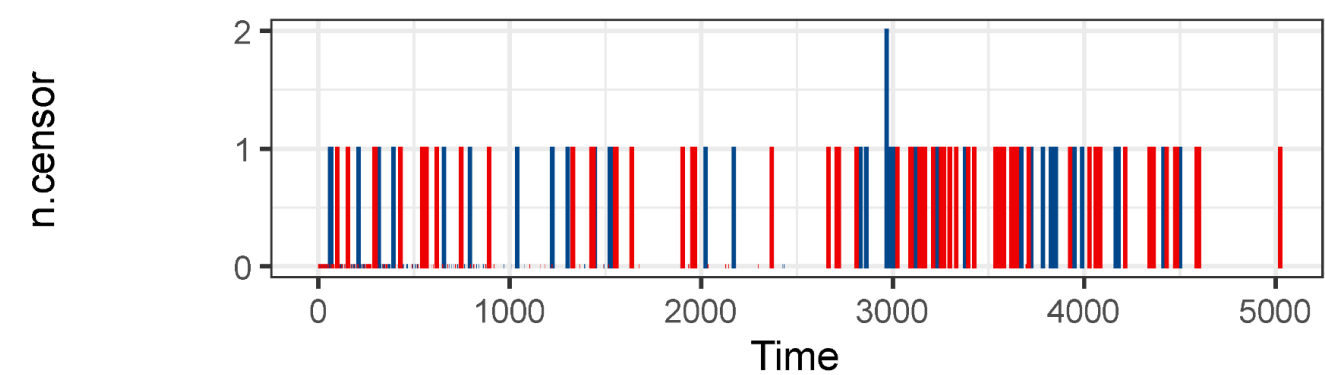

Supplement: Supplementary file 9 — Supplementary Figure 2. [file 41598_2021_94128_MOESM9_ESM.pdf]

A

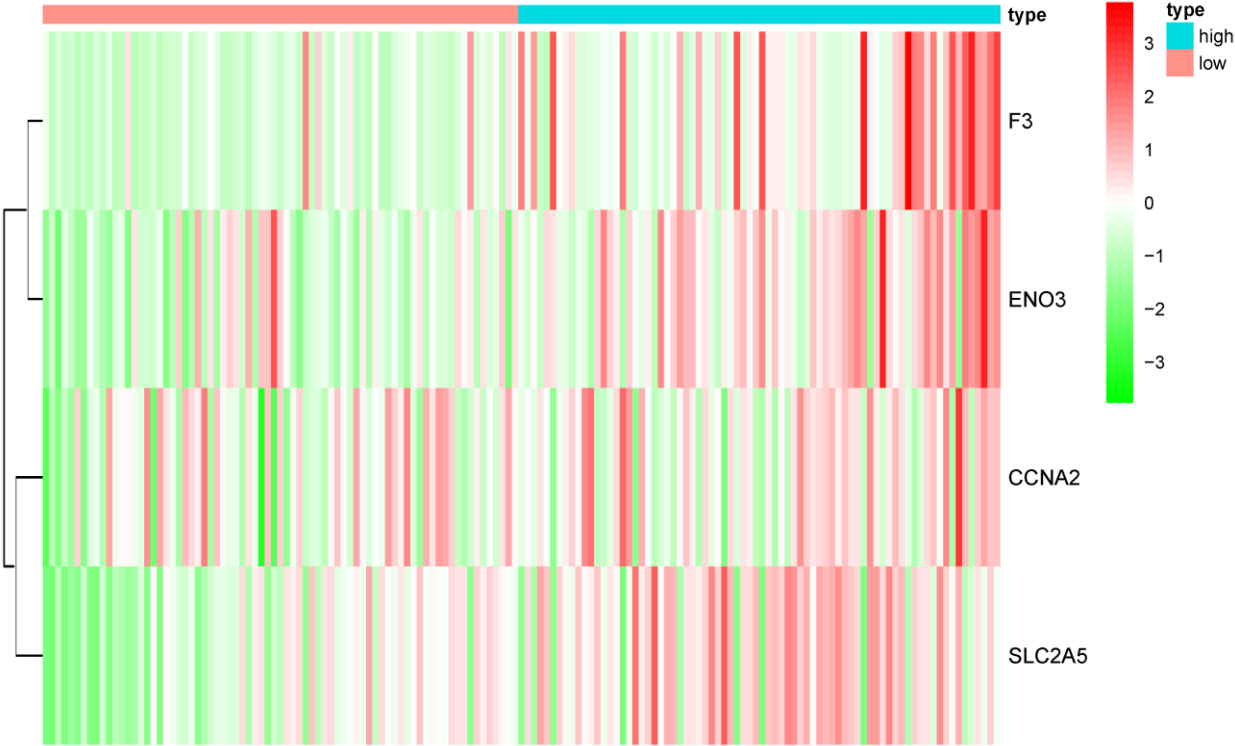

B

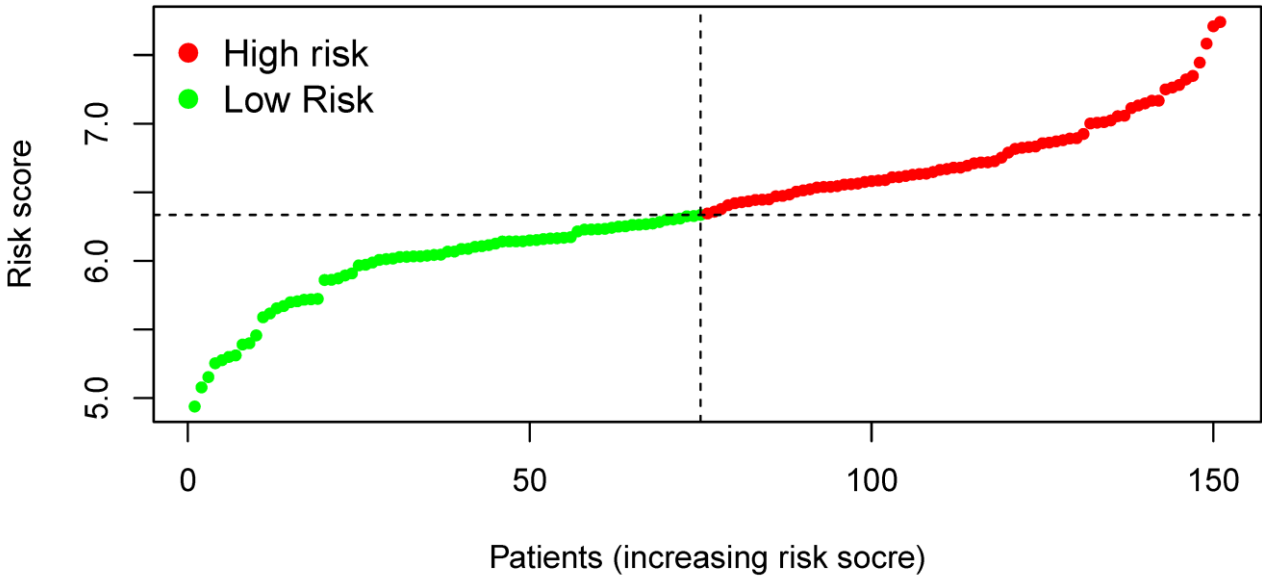

C

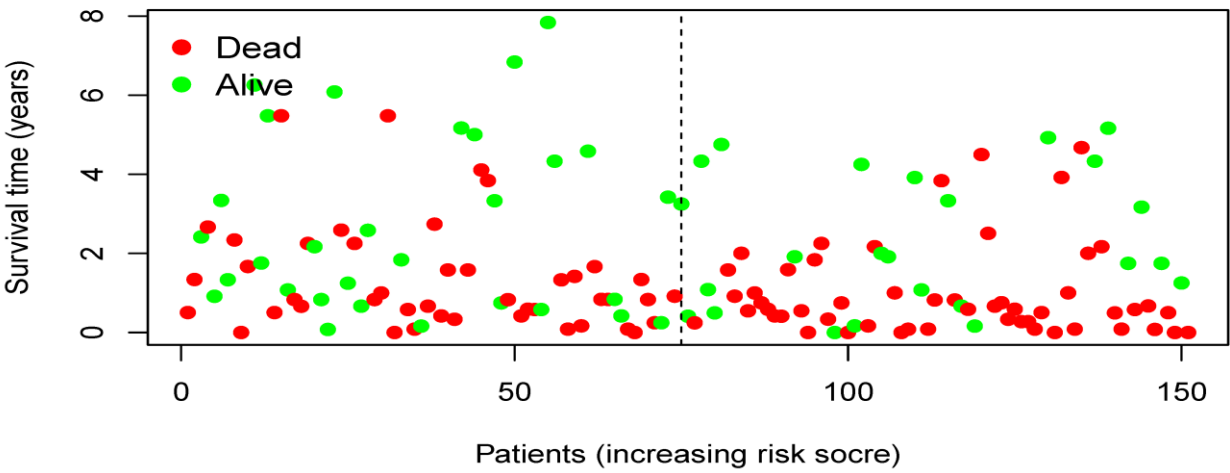

D

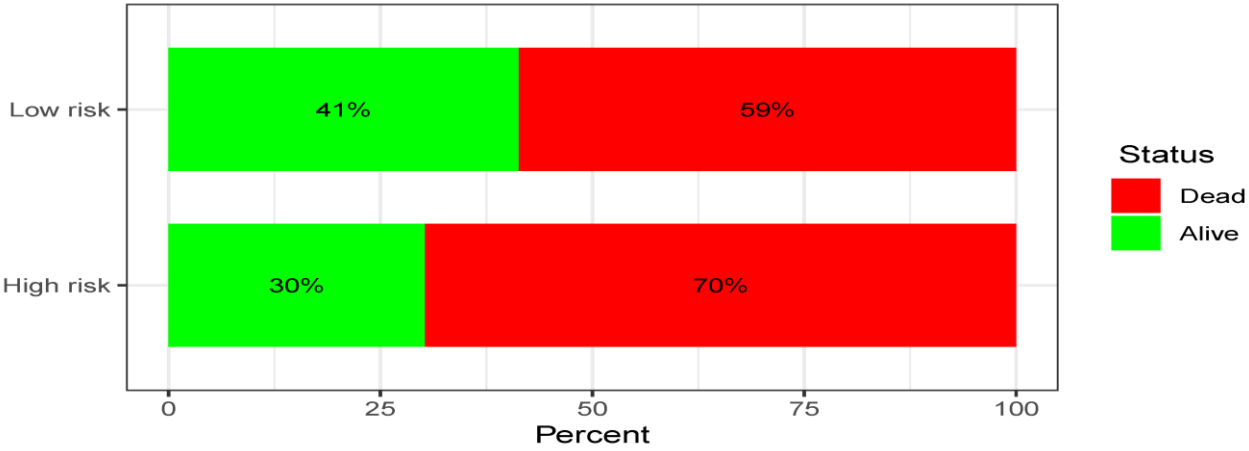

E

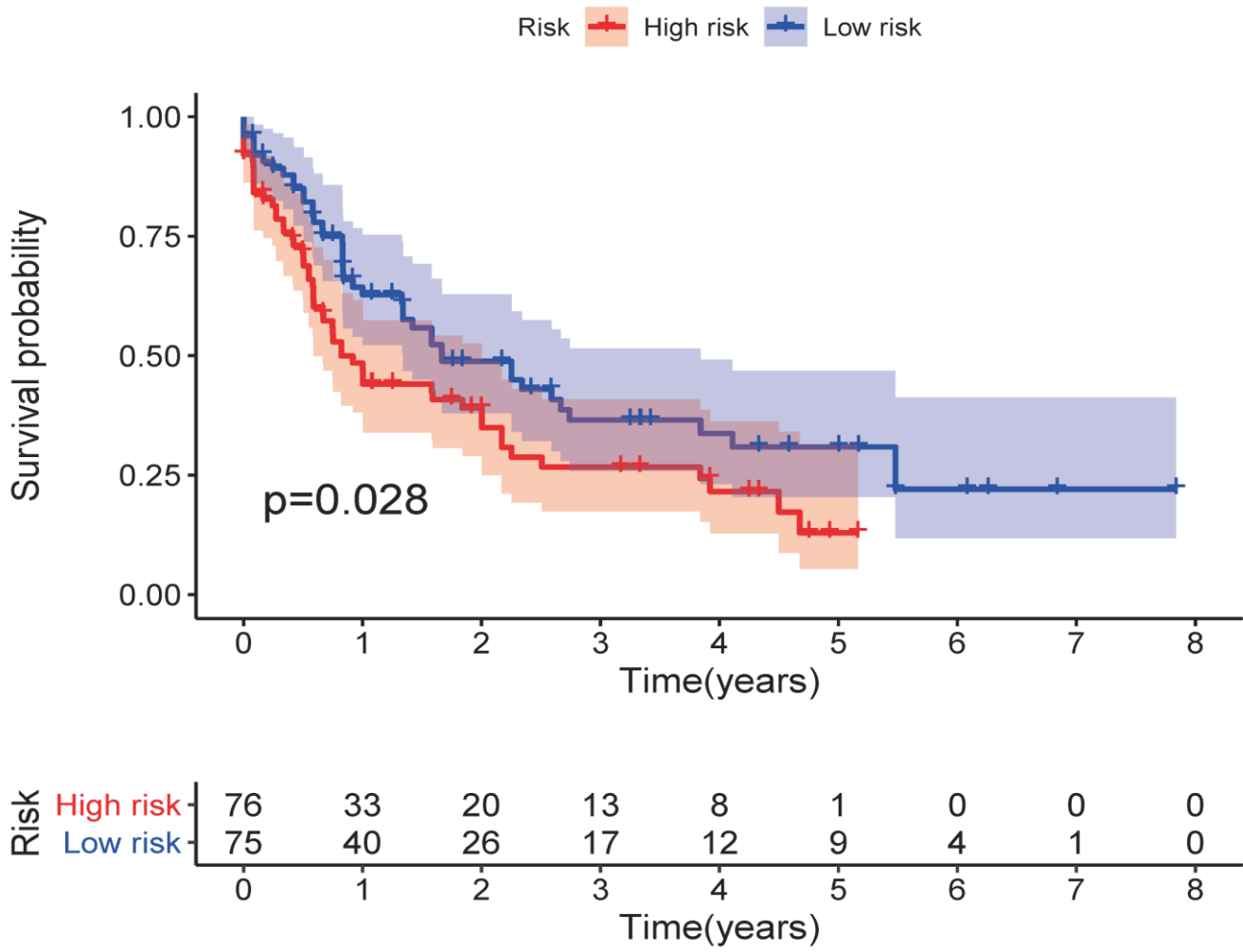

Supplement: Supplementary file 10 — Supplementary Figure 3. [file 41598_2021_94128_MOESM10_ESM.pdf]

A

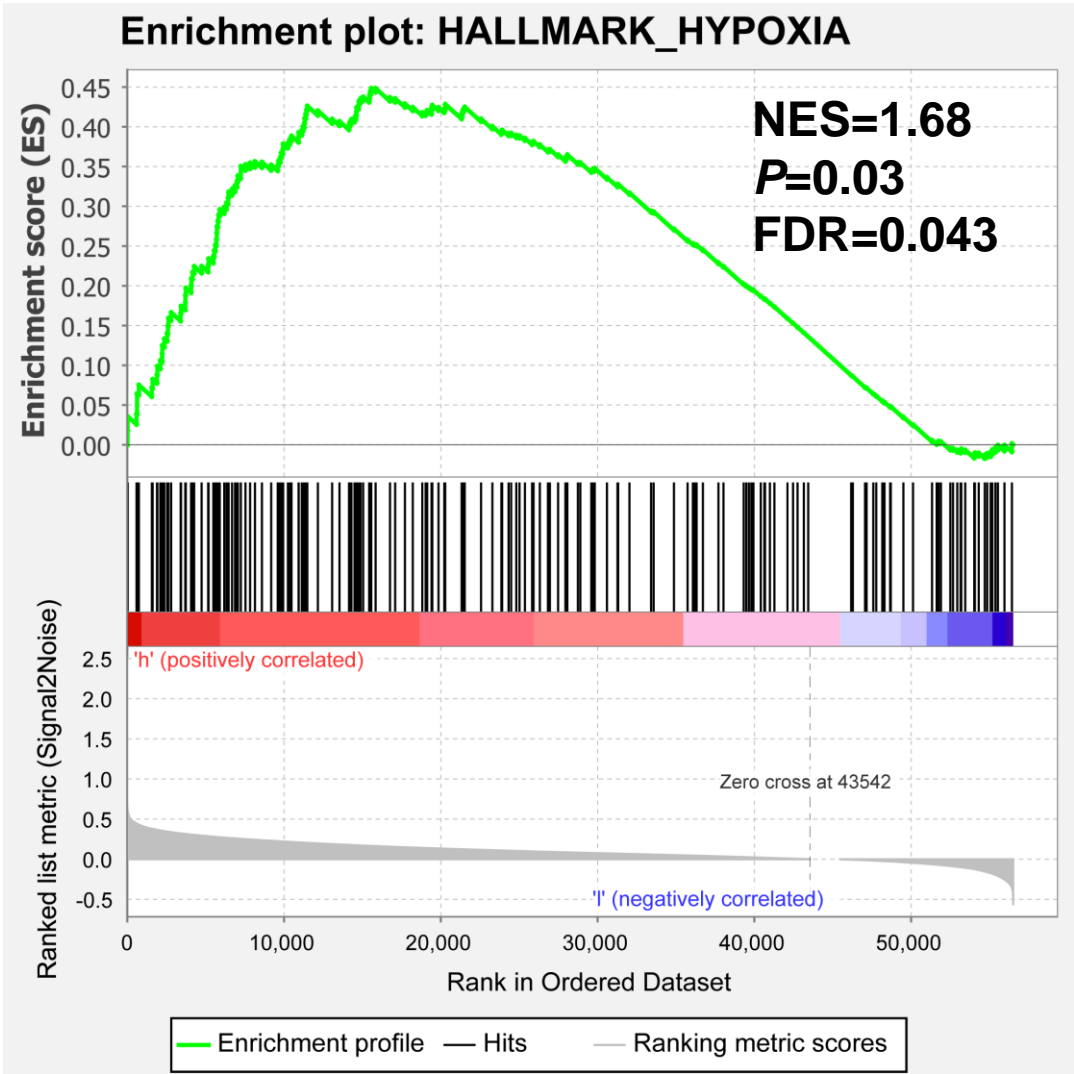

B

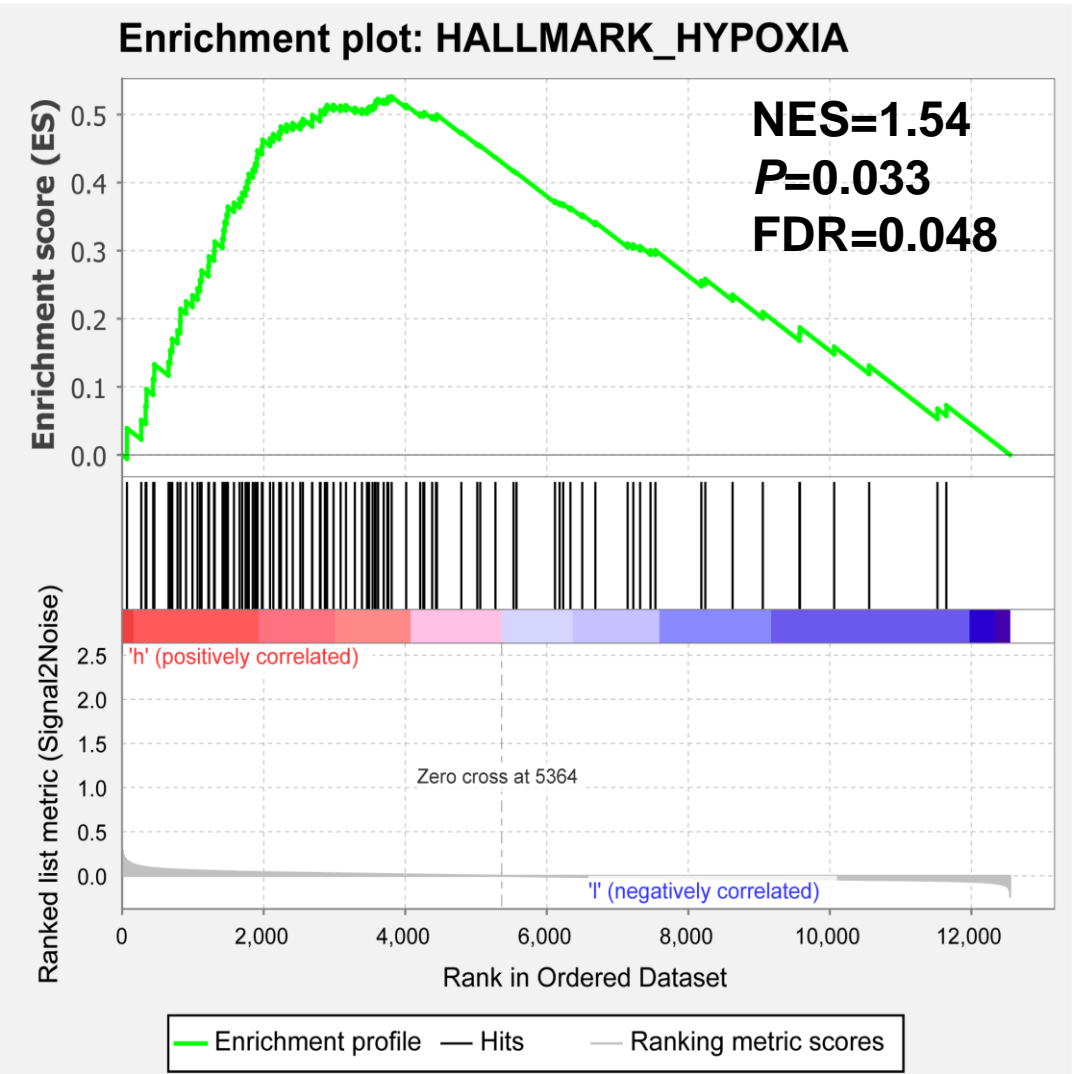

Supplement: Supplementary file 11 — Supplementary Figure 4. [file 41598_2021_94128_MOESM11_ESM.pdf]
